# Exploring potential associations between blood metabolites and cirrhosis risk: a Mendelian randomization and LC–MS/MS analysis

**DOI:** 10.3389/fmed.2026.1809188

**Published:** 2026-07-02

**Authors:** Duoduo Lv, Ning Han, Hong Tang

**Affiliations:** 1 Center of Infectious Diseases, West China Hospital of Sichuan University, Chengdu, Sichuan, China; 2 Laboratory of Infectious and Liver Diseases, Institution of Infectious Diseases, West China Hospital of Sichuan University, Chengdu, Sichuan, China; 3 Division of Infectious Diseases, State Key Laboratory of Biotherapy, Sichuan University, Chengdu, Sichuan, China

**Keywords:** Mendelian randomization, LC–MS/MS, cirrhosis, glutamine, metabolites

## Abstract

**Background:**

The development of cirrhosis is closely intertwined with metabolic processes. No previous studies have reported a clear causal relationship between cirrhosis and metabolic processes. Thus, this study aimed to explore the potential associations between blood metabolites and cirrhosis using Mendelian randomization (MR) combined with targeted metabolomics analysis.

**Methods:**

A two-sample MR analysis was conducted using genome-wide association study data to evaluate the associations of circulating metabolites with cirrhosis. Statistical evaluations employed inverse variance-weighted models, MR-Egger regression, and sensitivity tests addressing pleiotropy and heterogeneity. In addition, blood samples were collected from 10 patients with liver cirrhosis and 10 healthy controls. Blood amino acid concentrations were measured using liquid chromatography–tandem mass spectrometry (LC–MS/MS) to provide preliminary clinical evidence supporting the MR-derived candidate metabolites.

**Results:**

The MR analysis found that increases in 11 metabolites/metabolic ratios were associated with elevated risks of liver cirrhosis, whereas increases in the remaining 3 metabolites were related to the prevention of the occurrence of liver cirrhosis. Among these candidates, genetically predicted higher glutamine degradant levels were associated with a lower risk of cirrhosis (OR = 0.877, 95% CI: 0.784–0.981, *p* = 0.022). Pathway analysis further suggested that arginine biosynthesis, proline metabolism, and nitrogen metabolism may be involved in metabolic alterations related to cirrhosis. The LC–MS/MS analysis showed lower glutamate and glutathione levels in patients with cirrhosis than in controls, providing preliminary support for altered glutamine-related metabolism in cirrhosis.

**Conclusion:**

This study provides suggestive MR evidence linking specific circulating metabolites to cirrhosis risk. In particular, glutamine-related metabolic alterations may be associated with susceptibility to cirrhosis. These findings provide exploratory insights into metabolite-related pathways that may contribute to cirrhosis development.

## 1 Introduction

Cirrhosis is a late-stage liver disease resulting from acute or chronic liver injury caused by hepatitis virus infection, alcohol abuse, obesity, and other factors, and it can progress to liver failure and hepatocellular carcinoma (HCC) (1). In 2019, cirrhosis accounted for 2.4% of global deaths, and effective pharmacological therapies remain lacking (2). As a major cause of morbidity and mortality worldwide, cirrhosis places a substantial burden on healthcare systems, underscoring the need for deeper mechanistic insights into its development and progression.

A healthy liver is central to systemic metabolic homeostasis, functioning in the metabolism, storage, and redistribution of various nutrients such as lipids, carbohydrates, and vitamins (3–5). Metabolites can serve not only as accessible biomarkers for disease diagnosis and prognosis, but also as potentially reversible and pharmacologically targetable mediators involved in cirrhosis progression (6, 7). Unlike inherited genetic variants, which are largely nonmodifiable, metabolites represent dynamic downstream products of gene and environment interactions and may therefore provide greater clinical and translational value. Metabolic dysregulation is increasingly recognized as a critical contributor to the pathogenesis of cirrhosis (8, 9). Recent advances in metabolomics now enable the systematic evaluation of altered pathways and intermediate metabolites that may influence cirrhosis risk. Although numerous studies have reported associations between metabolic profiles and liver diseases, the potential role of individual metabolites in cirrhosis remains unclear (10, 11). Addressing this gap could open avenues for targeted strategies involving metabolic pathways to prevent or alleviate cirrhosis.

Mendelian randomization (MR) offers an approach for inferring causal relationships between risk factors and diseases, leveraging genetic variants as instrumental variables to minimize confounding and reverse causation bias (12). In cirrhosis research, MR may help explore whether specific metabolites are associated with disease risk, providing clues for future biomarker discovery and mechanistic studies. Recently, Chen et al. conducted a genome-wide association study (GWAS) that integrated 1,091 metabolites and 309 metabolite ratios, demonstrating associations between genetically determined metabolites and 12 diseases, including Parkinson’s disease, osteoarthritis, ischemic stroke, inflammatory bowel disease, and multiple sclerosis (13). However, research specifically addressing the relationship between metabolites and cirrhosis remains limited. In this study, we applied MR analysis to investigate potential links between serum metabolites and cirrhosis risk, aiming to provide novel insights into metabolism-related alterations involved in cirrhosis.

Recently, metabolomics has shown great potential in revealing the mechanisms of liver disease development and identifying biomarkers for clinical diagnosis (14–16). However, most findings to date have been based on observational data, making it difficult to determine whether metabolic changes are a cause or a consequence of cirrhosis. To address this gap, we combined MR with targeted liquid chromatography–tandem mass spectrometry (LC–MS/MS) analysis. By integrating genetic evidence with preliminary clinical observations of blood amino acids, we aimed to identify specific metabolites that may influence cirrhosis risk and provide new insights into the underlying disease mechanisms.

## 2 Materials and methods

### 2.1 Mendelian randomization

#### 2.1.1 Study design and data source

**Figure 1:**
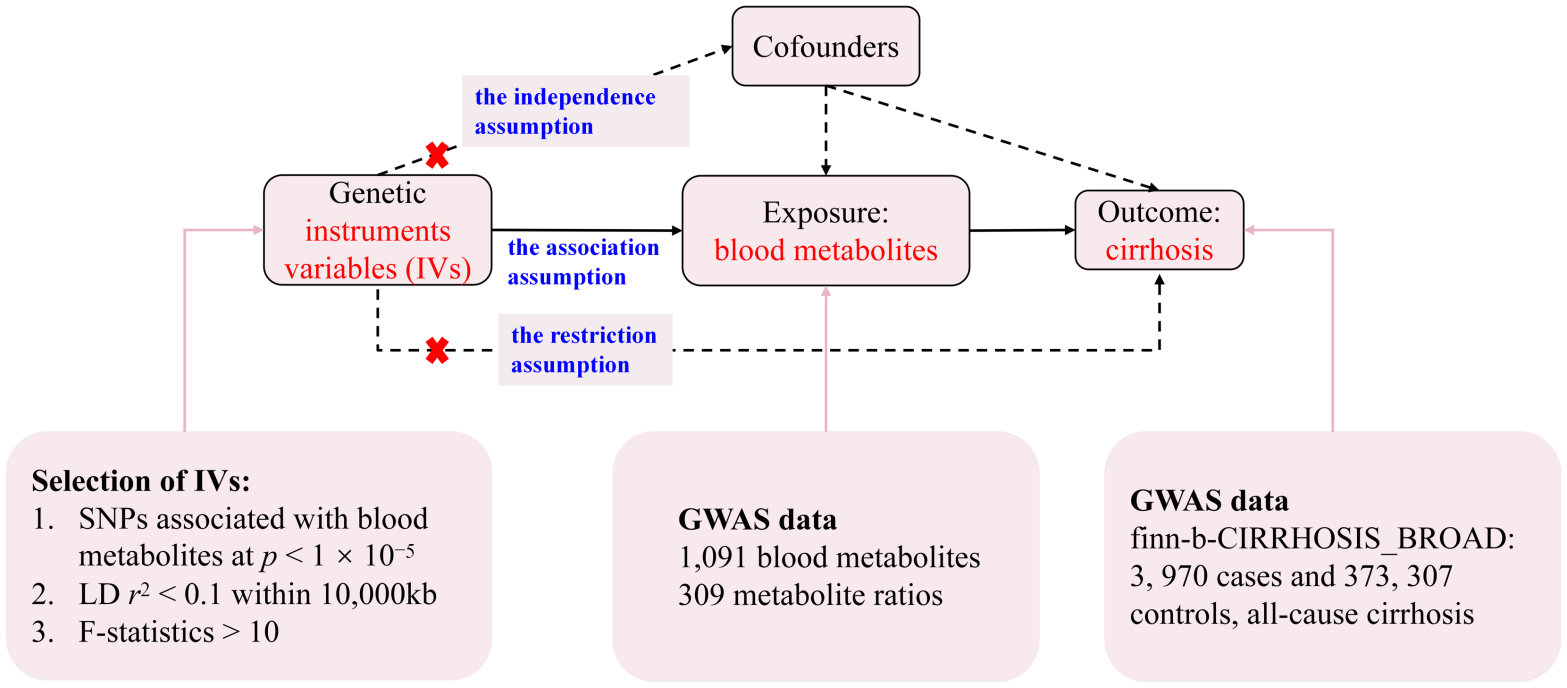
Research framework diagram. SNP, single-nucleotide polymorphism; LD, linkage disequilibrium; GWAS, genome-wide association study.

We conducted MR analyses using publicly available GWAS datasets to evaluate associations between genetically predicted blood metabolites and cirrhosis risk. In the primary analysis, blood metabolites were considered as exposures and cirrhosis was considered as the outcome. The MR framework adhered to the following three core assumptions: (1) Relevance: genetic instrumental variables are strongly associated with blood metabolites; (2) Exclusion restriction: instrumental variables influence cirrhosis only through metabolites and not via alternative pathways; and (3) Independence: instrumental variables are uncorrelated with potential confounders. To ensure rigor, instrumental variables were selected according to strict quality-control criteria, as illustrated in the flowchart presented in Figure 1.

Chen et al. (13) conducted a large-scale metabolomic analysis of 8,299 individuals from the Canadian Longitudinal Study of Aging, profiling plasma metabolites and performing GWAS. This remains the most comprehensive GWAS of metabolites to date, covering 1,091 plasma metabolites and 309 metabolite ratios, which served as the source of our instrumental variables. Cirrhosis data were obtained from 3,920 patients and 373,307 controls in the FINN-B-CIRRHOSIS_BROAD cohort, encompassing all-cause cirrhosis and primarily of European ancestry (17). Importantly, no overlap existed between the exposure and outcome datasets, supporting the reliability of our analysis.

#### 2.1.2 Selection of instrumental variables

To identify eligible single nucleotide polymorphisms (SNPs), we applied systematic screening thresholds. A lenient significance threshold (*p* < 1 × 10^−5^) was adopted to capture SNPs strongly associated with serum metabolites (18). To mitigate linkage disequilibrium (LD), we enforced *r*^2^ < 0.1 within a 10,000 kb window (19, 20). Instrumental variables with *F*-statistics > 10 were retained to minimize weak instrumental bias (21). Potential confounders such as smoking, alcohol consumption, physical activity, education, and diet, were excluded based on searches performed in the Phenotype Scanner V2.0 database (22).

#### 2.1.3 MR analyses

MR analyses were performed on 1,091 metabolites and 309 ratios using the TwoSampleMR package in R version 4.2.3. The primary analysis employed the inverse variance-weighted (IVW) method, which assumes all SNPs are valid instruments. Complementary analyses with MR-Egger regression and weighted median (WM) were used to evaluate the consistency of the MR estimates. Significance was defined as *p* < 3.5 × 10^−5^ (Bonferroni correction: 0.05/1,400 metabolites). Moreover, results with *p*-values of 3.5 × 10^−5^ to 0.05 were considered nominally significant. Following previous recommendations, candidate associations were retained when supported by at least two of the three MR methods (20). Sensitivity analyses included Cochran’s Q-test for heterogeneity, MR-Egger intercept and MR-Pleiotropy RESidual Sum and Outlier (MR-PRESSO) for pleiotropy, and both SNP-specific effect estimates and leave-one-out (LOO) analyses to identify influential variants.

To explore potential reverse causation, we further performed reverse MR analyses after identifying the candidate metabolites in the forward analysis. Cirrhosis was treated as the exposure, and the candidate metabolites were treated as outcomes. The reverse analyses used the same MR framework, with the IVW method as the primary method and WM and MR-Egger as complementary sensitivity methods.

#### 2.1.4 Metabolic pathway analysis

Pathway analysis of the identified metabolites was conducted using MetaboAnalyst 6.0 in conjunction with the KEGG database, providing functional interpretation of the results (23).

### 2.2 LC–MS/MS analysis

#### 2.2.1 Participants

A total of 10 patients with non-alcoholic cirrhosis (7 men and 3 women; mean age, 47 years) were enrolled from West China Hospital, Sichuan University, China. In addition, 10 healthy volunteers (6 men and 4 women; mean age, 34 years) were included as controls. The study was approved by the Ethics Committee of our institution, and written informed consent was obtained from all participants prior to enrollment. The severity of liver disease was staged according to the Child-Pugh classification: seven patients were classified as Child-Pugh class A and three as Child-Pugh class B (Supplementary Table S1).

#### 2.2.2 Blood sample

Blood samples were collected from all participants in the morning after an overnight fast using heparin-containing vacutainers. Plasma was separated by centrifugation at 2,000 *g* for 10 min at 4 °C and stored at −80 °C. The samples were mixed with an acetonitrile/methanol (1:1) mixture containing internal standards by thorough vortexing. After ultrasonication for 10 min, the samples were incubated at −20 °C for 60 min and then centrifuged at 12,000 rpm for 10 min. The supernatant was diluted and centrifuged at 12,000 rpm for 10 min. Finally, the supernatant was injected into the LC–MS/MS system for analysis.

#### 2.2.3 LC–MS/MS analysis procedure

An ultra-high performance LC–MS/MS system (ExionLC™ AD UHPLC-QTRAP 6500+, AB SCIEX Corp., Boston, MA, USA) was used to quantify amino acids at Novogene Co., Ltd. (Beijing, China). All 136 amino acid standards and 5 stable isotope-labeled standards were obtained from Sigma-Aldrich (St. Louis, MO, USA) and ZZ Standards Co., Ltd. (Shanghai, China). Separation was performed on an ACQUITY UPLC BEH Amide column (2.1 × 100 mm, 1.7 μm) which was maintained at 50 °C. The mobile phase, consisting of 0.1% formic acid in 5 mM ammonium acetate (solvent A) and 0.1% formic acid in acetonitrile (solvent B), was delivered at a flow rate of 0.30 mL/min. The solvent gradient was set as follows: initial 90% B, 1.0 min; 90–85% B, 2.0 min; 90–85% B, 2.0 min; 85–75% B, 3.5 min; 75–70% B, 7.0 min; 70–45% B, 10.0 min; 45–90% B, 11.1 min; and 90% B, 13.0 min. The mass spectrometer was operated in positive multiple reaction monitoring mode. The parameters were as follows: ion spray voltage, 5,500 V; curtain gas, 35 psi; ion source temperature, 550 °C; and ion source gases 1 and 2 (50 and 60 psi).

The limit of detection (LOD) and limit of quantification (LOQ) was determined using the method of signal-to-noise ratio (S/N), which compares the signal measured by the standard solution concentration with the blank matrix. Generally, concentrations corresponding to S/N ratios of 3:1 and 10:1 were defined as the LOD and LOQ, respectively. Quality control (QC) samples were inserted at regular intervals during LC–MS/MS analysis, and analytical stability was evaluated by calculating the relative standard deviation of target analyte concentrations in all QC samples based on standard calibration curves, with relative standard deviation ≤ 15% considered acceptable. Peak areas were normalized using corresponding internal standards, and QC-based signal correction was applied to reduce analytical drift when necessary. Metabolites with unreliable detection, concentrations below the LOQ, or missing values beyond predefined QC criteria were excluded from further analyses.

#### 2.2.4 Statistical analysis

The data on amino acid concentrations were statistically analyzed and expressed as mean ± SD. The normality of metabolite distributions was assessed using the Shapiro–Wilk test. Differences between the cirrhosis and control groups were analyzed using the independent samples *t*-test for normally distributed data or the Mann–Whitney *U* test for nonnormally distributed data. and that *p* < 0.05 was considered nominally significant. All analyses were performed using R version 4.2.

## 3 Results

### 3.1 MR analysis of blood metabolites and cirrhosis

#### 3.1.1 Strength of the instrumental variables

Based on instrument selection, 1,059 metabolites and 293 metabolite ratios were retained (Supplementary Table S2). Forty-eight metabolites were excluded because they had fewer than three SNPs, as multiple independent instruments were required for reliable MR estimation and sensitivity analyses. All retained SNPs had *F*-statistics > 10, indicating the absence of weak instruments (Supplementary Table S3). Harmonized data are provided in Supplementary Table S2.

#### 3.1.2 MR analysis

**Figure 2:**
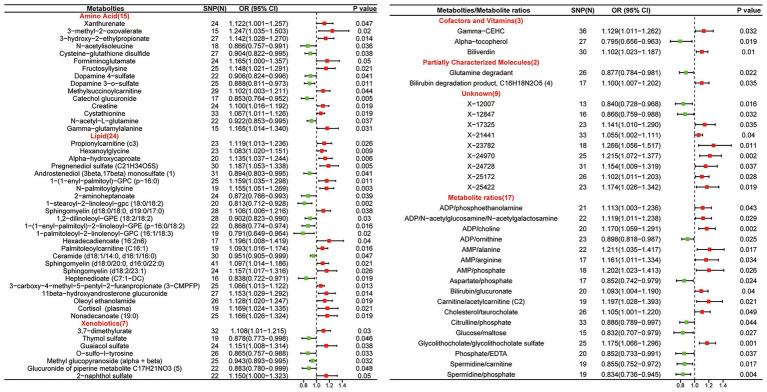
Comprehensive forest plot of the 77 metabolites and metabolite ratios nominally associated with cirrhosis risk in the IVW analysis. Green dots represent a negative correlation with cirrhosis risk, whereas red dots indicate a positive correlation. CI, confidence interval; IVW, inverse variance-weighted; OR, odds ratio.

**Figure 3:**
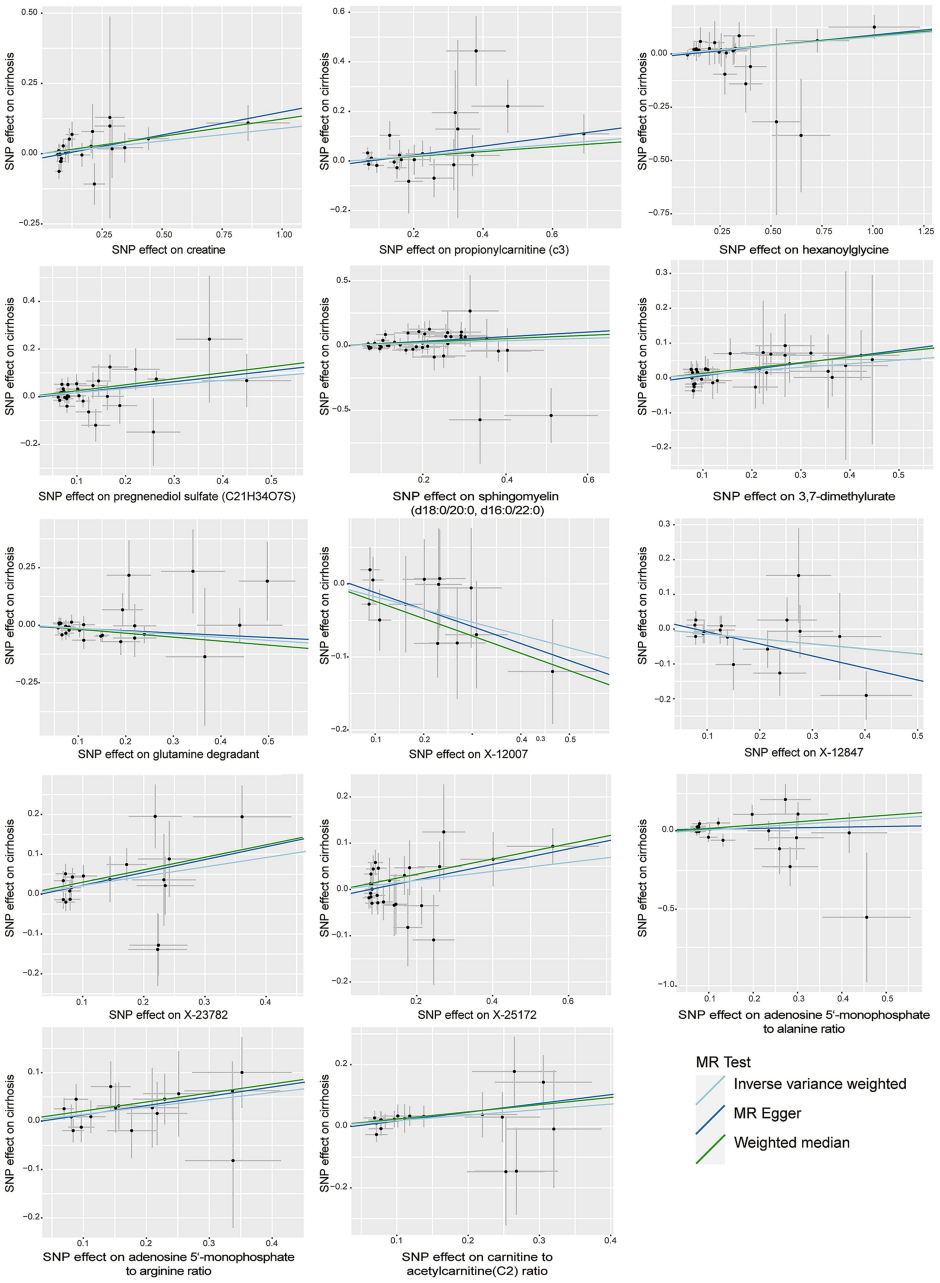
Scatter plots showing significant Mendelian randomization associations (*p* < 0.05) between metabolites and cirrhosis. SNP, single nucleotide polymorphism.

Figure 2 presents the 77 metabolites/metabolite ratios that were nominally associated with cirrhosis risk in the IVW analysis, of which 9 remained chemically uncharacterized. The identified set comprised 15 amino acids, 24 lipids, 7 xenobiotics, 3 cofactors/vitamins, 2 partially characterized molecules, and 17 metabolite ratios. Applying the selection criterion that at least two of the three MR methods showed directionally consistent nominal associations, 14 metabolites/metabolite ratios were retained as candidates (Table 1 and Figure 3).

**Table 1:** MR analysis of the causal association between blood metabolites and cirrhosis.

Metabolites	SNP (*N*)	WM		MR-Egger		IVW	
		OR (95% CI)	*p*-value	OR (95% CI)	*p*-value	OR (95% CI)	*p*-value
Amino acid (1)							
Creatine	24	1.131 (1.013, 1.263)	0.028	1.177 (1.045, 1.327)	0.014	1.100 (1.016–1.192)	0.019
Lipid (4)							
Propionylcarnitine (C3)	23	1.101 (0.956, 1.268)	0.182	1.201 (1.014, 1.423)	0.046	1.119 (1.013–1.236)	0.026
Hexanoylglycine	23	1.088 (1.003, 1.180)	0.041	1.099 (0.993, 1.216)	0.082	1.083 (1.020–1.151)	0.009
Pregnenediol sulfate (C_21_H_34_O_5_S)	30	1.286 (1.085, 1.524)	0.004	1.259 (0.954, 1.660)	0.114	1.187 (1.053–1.338)	0.005
Sphingomyelin (d18:0/20:0, d16:0/22:0)	41	1.140 (1.013, 1.282)	0.029	1.176 (1.005, 1.377)	0.049	1.097 (1.014–1.186)	0.021
Xenobiotics (1)							
3,7-dimethylurate	32	1.162 (1.023, 1.320)	0.021	1.200 (1.000, 1.441)	0.059	1.108 (1.010–1.215)	0.030
Partially characterized molecules (1)							
Glutamine degradant	26	0.842 (0.713, 0.993)	0.041	0.907 (0.726, 1.132)	0.398	0.877 (0.784–0.981)	0.022
Unknown (4)							
X-12007 levels	13	0.789 (0.649, 0.959)	0.017	0.792 (0.602, 1.042)	0.124	0.840 (0.728–0.968)	0.016
X-12847 levels	16	0.865 (0.712, 1.051)	0.144	0.701 (0.528, 0.932)	0.028	0.866 (0.759–0.988)	0.032
X-23782 levels	18	1.363 (1.110, 1.675)	0.003	1.379 (0.912, 2.085)	0.148	1.266 (1.056–1.517)	0.011
X-25172 levels	26	1.179 (1.033, 1.346)	0.015	1.183 (1.028, 1.361)	0.028	1.102 (1.011–1.203)	0.028
Metabolite ratios (3)							
AMP-to-alanine ratio	22	1.234 (1.015, 1.499)	0.035	1.032 (0.715, 1.489)	0.868	1.211 (1.035–1.417)	0.017
AMP-to-arginine ratio	17	1.204 (1.006, 1.441)	0.042	1.205 (0.908, 1.597)	0.216	1.161 (1.011–1.334)	0.034
Carnitine-to-acetylcarnitine (C2) ratio	19	1.263 (1.023, 1.558)	0.030	1.329 (0.899, 1.966)	0.172	1.197 (1.028–1.393)	0.021

AMP, adenosine 5′-monophosphate; CI, confidence interval; IVW, inverse variance-weighted; OR, odds ratio; WM, weighted median.

As shown in Table 1, 11 metabolites/metabolite ratios were positively associated with cirrhosis risk, including creatine (IVW odds ratio [OR] = 1.100; 95% confidence interval [CI] = 1.016–1.192; *p* = 0.019), propionylcarnitine (C3) (OR = 1.119; 95% CI = 1.013–1.236; *p* = 0.026), hexanoylglycine (OR = 1.083; 95% CI = 1.020–1.151; *p* = 0.009), pregnenediol sulfate (C_21_H_34_O_5_S) (OR = 1.187; 95% CI = 1.053–1.338; *p* = 0.005), sphingomyelin (d18:0/20:0, d16:0/22:0) (OR = 1.097; 95% CI = 1.014–1.186; *p* = 0.021), 3,7-dimethylurate (OR = 1.108; 95% CI = 1.101–1.215; *p* = 0.030), X-23782 (OR = 1.266; 95% CI = 1.056–1.517; *p* = 0.011), X-25172 (OR = 1.102; 95% CI = 1.011–1.203; *p* = 0.028), AMP-to-alanine ratio (OR = 1.211; 95% CI = 1.035–1.417; *p* = 0.017), AMP-to-arginine ratio (OR = 1.161; 95% CI = 1.011–1.334; *p* = 0.034), and carnitine-to-acetylcarnitine (C2) ratio (OR = 1.197; 95% CI = 1.028–1.393; *p* = 0.021). Conversely, three metabolites were negatively associated with cirrhosis risk: glutamine degradant (OR = 0.877; 95% CI = 0.784–0.981; *p* = 0.022), X-12007 (OR = 0.840; 95% CI = 0.728–0.968; *p* = 0.016), and X-12847 (OR = 0.866; 95% CI = 0.759–0.988; *p* = 0.032). None of the 14 metabolites/metabolite ratios reached Bonferroni significance, but all met the relaxed suggestive threshold (3.5 × 10^−5^ < *p* < 0.05). The estimates from the IVW, WM, and MR-Egger methods were generally directionally consistent, supporting the robustness of these suggestive associations.

#### 3.1.3 Sensitivity analysis

Sensitivity analyses, including Cochran’s *Q* test, MR-Egger regression, MR-PRESSO, LOO analysis, and funnel plots, demonstrated no evidence of heterogeneity or horizontal pleiotropy. MR-PRESSO did not detect outliers (*p* > 0.05; Table 2), and the LOO analysis excluded any single SNP with disproportionate influence (Supplementary Figures S1–S14). PhenoScanner analysis confirmed no associations with known confounders.

**Table 2:** Sensitivity analysis of the causal association between blood metabolites and cirrhosis.

Metabolites	SNP (*N*)	Heterogeneity		Pleiotropy		MR-PRESSO *p*-value
		Cochran’s *Q* value	*p-*value	MR-Egger intercept	*p-*value	
Amino acid (1)						
Creatine	24	19.309	0.683	−0.015	0.149	0.754
Lipid (4)						
Propionylcarnitine (C3)	23	21.833	0.470	−0.013	0.327	0.513
Hexanoylglycine	23	10.323	0.983	−0.005	0.733	0.985
Pregnenediol sulfate (C_21_H_34_O_5_S)	30	32.928	0.281	−0.007	0.648	0.301
Sphingomyelin (d18:0/20:0, d16:0/22:0)	41	42.266	0.373	−0.012	0.320	0.387
Xenobiotics (1)						
3,7-dimethylurate	32	16.667	0.983	−0.012	0.328	0.984
Partially characterized molecules (1)						
Glutamine degradant	26	20.152	0.739	−0.004	0.732	0.761
Unknown (4)						
X-12007 levels	13	5.559	0.937	0.011	0.637	0.948
X-12847 levels	16	14.653	0.477	0.032	0.123	0.453
X-23782 levels	18	26.017	0.074	−0.010	0.657	0.107
X-25172 levels	26	24.689	0.480	−0.013	0.224	0.487
Metabolite ratios (3)						
AMP to alanine ratio	22	26.746	0.179	0.018	0.355	0.182
AMP to arginine ratio	17	7.271	0.968	−0.006	0.775	0.971
Carnitine to acetylcarnitine (C2) ratio	19	10.243	0.924	−0.011	0.576	0.940

AMP, adenosine 5′-monophosphate; MR, Mendelian randomization; PRESSO, pleiotropy residual sum and outlier; SNP, single-nucleotide polymorphism.

#### 3.1.4 Reverse MR analysis

To explore potential reverse causation, we performed reverse MR using cirrhosis as the exposure and the candidate metabolites as outcomes. After harmonization, three cirrhosis-associated instruments were retained for each candidate metabolite. The reverse analyses did not show a consistent pattern suggesting that genetically predicted cirrhosis broadly influenced the candidate metabolites. A nominal reverse association was observed for sphingomyelin (d18:0/20:0, d16:0/22:0) levels in the IVW analysis (*β* = −0.093, SE = 0.039, *p* = 0.017), whereas no nominal reverse association was observed for glutamine degradant or most other candidates. MR-Egger intercept tests did not indicate significant directional pleiotropy. The reverse MR estimates and sensitivity analyses are provided in Supplementary Table S4.

#### 3.1.5 Metabolic pathway analysis

As four of the identified metabolites were uncharacterized, pathway enrichment analysis was conducted for the remaining 10 metabolites. For comparisons in Table 3, nominal *p*-values are presented. Multiple-comparison adjustment was additionally performed using the Benjamini–Hochberg false discovery rate (FDR) method, and no pathway remained statistically significant after FDR correction. Therefore, this analysis identified five potentially relevant metabolic pathways (Table 3), including arginine biosynthesis, proline metabolism, and nitrogen metabolism. Additionally, 3,7-dimethylurate was mapped to caffeine metabolism (*p* = 0.044).

**Table 3:** Significant metabolic pathways involved in the pathogenesis of cirrhosis.

Pathway name	Involved metabolites/ratios	Nominal *p*-value
Arginine biosynthesis	Glutamine degradant/AMP-to-arginine ratio	0.002
Arginine and proline metabolism	Creatine/AMP-to-arginine ratio	0.010
Nitrogen metabolism	Glutamine degradant	0.026
Purine metabolism	AMP-to-alanine ratio/AMP-to-arginine ratio	0.035
Caffeine metabolism	3,7-dimethylurate	0.044

AMP, adenosine 5′-monophosphate.

### 3.2 Preliminary clinical analysis: glutamine and its metabolites

**Figure 4:**
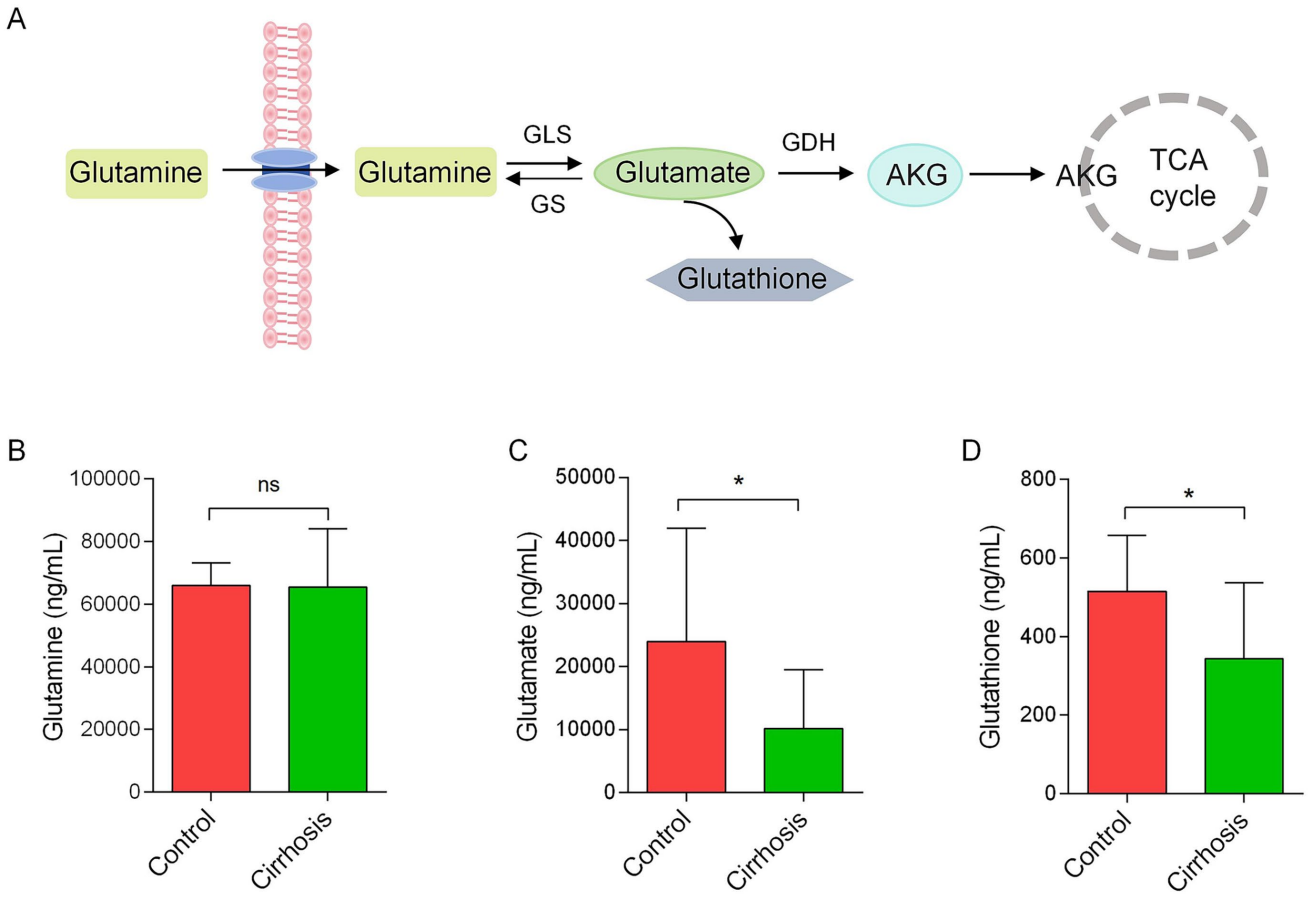
Comparison of glutamine and its metabolite concentration levels in plasma. **(A)** Pathways of glutamine metabolism. Glutamine metabolism in the body can be converted into glutamate through the deamination reaction catalyzed by GLS. Glutamate is further converted by GDH into AKG, an intermediate of the TCA cycle. Another, glutamate in the cytoplasm can produce glutathione. **(B)**, **(C)** and **(D)**. Statistical Plots of blood levels of Glutamine, glutamate, and glutathione levels in control and cirrhosis groups. AKG, alpha-ketoglutarate; GLS, glutaminase; GDH, glutamate dehydrogenase; GS, glutamine Synthetase; TCA, tricarboxylic acid. *p<0.05.

To provide preliminary clinical support for MR-derived candidates, we performed targeted quantification of plasma amino acids in a clinical cohort. LC–MS/MS analysis revealed no significant difference in average plasma glutamine concentrations between patients with cirrhosis (65,997 ± 2,268 ng/mL) and healthy controls (65,477 ± 5,908 ng/mL; Figure 4B). This finding suggests that plasma glutamine levels may remain relatively stable in this small clinical cohort.

As illustrated in Figure 4A, glutamine serves as a critical precursor for glutamate and glutathione synthesis, which are vital for cellular metabolism and redox balance. Further investigation into glutamine degradants, including relevant downstream metabolites such as glutamate and glutathione, revealed a marked divergence from stable glutamine levels. In patients with liver cirrhosis, the concentrations of both glutamate and glutathione were lower than those in healthy controls (Figures 4C,D). These preliminary LC–MS/MS findings may provide supportive biochemical context for the MR results, in which genetically predicted higher glutamine degradant levels were associated with a lower risk of cirrhosis (OR = 0.877). Additionally, our descriptive analysis showed no apparent difference in glutamine and glutathione levels between patients with Child-Pugh A and B cirrhosis. By contrast, glutamate levels tended to be lower in patients with Child-Pugh B cirrhosis (Supplementary Figure S15). Given the small sample size and age imbalance of the clinical cohort, these clinical results should be interpreted cautiously. Together, these findings suggest that glutamine-related metabolic alterations, rather than glutamine concentration alone, may be associated with cirrhosis risk.

## 4 Discussion

In our MR analysis, genetic variants were used as instrumental variables to infer the potential associations between circulating metabolites and cirrhosis risk, whereas the biologically relevant exposures of interest were the metabolites themselves. Metabolites with fewer than three instrumental SNPs were excluded to improve the reliability of MR estimates and reduce the influence of single-variant-driven results. Our analysis identified 14 metabolites/metabolite ratios with suggestive, genetically predicted associations with cirrhosis risk. Compared with static genetic susceptibility loci, metabolite-related pathways may provide more actionable insights for biomarker development, therapeutic targeting, and clinical intervention in cirrhosis (24). Elevated levels of 11 metabolites/metabolite ratios, including creatine, propionylcarnitine (C3), hexanoylglycine, pregnenediol sulfate (C_21_H_34_O_5_S), sphingomyelin (d18:0/20:0, d16:0/22:0), 3,7-dimethylurate, X-23782, X-25172, AMP-to-alanine ratio, AMP-to-arginine ratio, and creatine-to-acetylcarnitine (C2) ratio, were associated with an increased risk of cirrhosis. In contrast, elevated levels of three metabolites, including a glutamine degradant, X-12007, and X-12847, were associated with reduced risks. We further identified five potential metabolic pathways implicated in the development of cirrhosis. To the best of our knowledge, this is one of the first MR studies to systematically assess potential associations between circulating metabolites and cirrhosis risk.

The high prevalence and mortality of cirrhosis place a considerable burden on patients and their families. Because cirrhosis is often asymptomatic in its early stages, it frequently remains undiagnosed until advanced disease develops, highlighting the critical importance of screening and prevention (25, 26). For example, lysosomal acid lipase activity is one of the strongest predictors of cirrhosis (area under the receiver operating characteristic curve = 0.859; *p* < 0.001) and may serve as a promising noninvasive marker (27). Moreover, a meta-analysis demonstrated significantly elevated adiponectin levels in patients with cirrhosis compared with controls (8.181; 95% CI = 3.676–12.686), suggesting its potential as a biomarker of advanced disease (28). Despite these findings, evidence clarifying potential links between metabolic dysregulation and cirrhosis remains insufficient. Building on the metabolite GWAS by Chen et al. (13), we designed this MR study to systematically evaluate metabolite–cirrhosis associations, with the goal of uncovering metabolite-related pathways and identifying potential targets for early detection and prevention.

Our study identified several metabolites showing positive associations with cirrhosis risk, some of which have been previously characterized. Creatine, a naturally occurring non-protein compound central to energy metabolism in muscle and brain tissues, is primarily degraded into creatinine (29). Evidence from the 2017–2018 National Health and Nutrition Examination Survey indicated the absence of a significant association between daily creatine intake (approximately 0.88 g/day) and the risk of liver fibrosis, cirrhosis, or fatty liver disease (30). By contrast, an animal study demonstrated that long-term, high-dose creatine supplementation in sedentary animals significantly elevated liver enzyme levels, leading to concomitant hepatic injury (31). Thus, the impact of creatine on cirrhosis remains unresolved and warrants further investigation. Previous metabolomic analyses have suggested that propionylcarnitine may serve as a diagnostic and prognostic biomarker for primary biliary cirrhosis and alcoholic hepatitis (32, 33), although its mechanistic role remains unclear. Sphingomyelin, a key membrane lipid, is metabolized to ceramide, which has been implicated in liver fibrosis progression (34). Likewise, animal studies have shown that elevated phosphatidylcholine levels are associated with exacerbated fibrosis and inflammation (35, 36), and these results are consistent with our findings. Moreover, metabolomic profiling has revealed significant dysregulation of caffeine and its metabolites in patients with alcohol-related liver disease (37). Our study extends these observations by suggesting that genetically predicted higher 3,7-dimethylurate levels may be associated with an increased risk of cirrhosis. By contrast, few studies have investigated the relationship between other identified metabolites and cirrhosis, highlighting the value of MR analysis in prioritizing metabolite candidates for further investigation.

Our study also identified several metabolites that were inversely associated with cirrhosis risk, including two that remained chemically uncharacterized. Notably, genetically predicted higher glutamine degradant levels were inversely associated with cirrhosis risk. Glutamine metabolism may play an important role in the biology of liver disease (38). Upon uptake, glutamine can be deamidated to glutamate, which serves as an important metabolic intermediate involved in protein synthesis and glutathione production (39). Given that the liver contributes to over 90% of systemic glutathione turnover and is the principal regulator of interorgan glutathione homeostasis (40), cirrhosis may disrupt endogenous glutathione synthesis and utilization. Indeed, previous evidence has shown that cirrhotic patients have lower plasma or erythrocyte glutathione levels than healthy controls (41, 42). These observations are broadly consistent with our MR findings, which suggest that genetically predicted higher glutamine degradant levels were associated with a lower risk of cirrhosis. Although alpha-ketoglutarate, another downstream product of glutamine metabolism, has been reported to suppress hepatic stellate cell activation *in vitro* and attenuate hepatic fibrosis *in vivo* without exacerbating hepatocyte injury (43, 44), it was not measured in our LC–MS/MS analysis. More importantly, because the MR exposure was annotated as “glutamine degradant” and its precise biochemical identity cannot be fully resolved from the GWAS annotation alone, the potential role of this pathway remains indirect and requires further experimental studies to clarify the underlying mechanisms.

This study has several notable strengths. First, it represents a comprehensive MR analysis of cirrhosis to date, encompassing 1,400 blood metabolites and metabolite ratios. Second, the use of multiple MR models and sensitivity analyses supported the robustness of the suggestive associations. Nevertheless, several limitations should be acknowledged. First, the instrument selection criterion may have excluded metabolites with less well-characterized genetic backgrounds, thereby limiting the overall coverage of the circulating metabolome. Reverse causation also remains possible because cirrhosis can alter systemic metabolism. Although exploratory reverse MR analyses did not show a consistent effect of genetically predicted cirrhosis on the candidate metabolites, these results should be interpreted cautiously given the limited number of instruments and the suggestive nature of the forward findings. Second, all GWAS datasets analyzed were derived from European populations, which may restrict generalizability. Moreover, the FinnGen outcome represented all-cause cirrhosis, whereas the LC–MS/MS clinical cohort included patients with non-alcoholic cirrhosis, and etiology-specific metabolic differences may limit direct comparability. Third, some metabolites identified as associated with cirrhosis remained chemically uncharacterized, precluding mechanistic interpretation. Future studies using targeted mass spectrometry, spectral library matching, and experimental confirmation are needed to determine their chemical identities and biological relevance of these metabolites. Moreover, although several downstream products of glutamine metabolism may be biologically involved in anti-fibrotic regulation, these metabolites were not quantitatively assessed in our LC–MS/MS clinical analysis, which limits further mechanistic interpretation of the glutamine metabolic axis in liver cirrhosis. Therefore, the LC–MS/MS findings should be interpreted as preliminary supportive evidence rather than definitive clinical validation. Larger age-matched cohorts with broader metabolomic coverage are needed to confirm our findings.

In conclusion, we identified 14 metabolites/metabolite ratios showing suggestive genetically predicted associations with cirrhosis risk, providing preliminary evidence for metabolite-related pathways involved in cirrhosis development. Specifically, our findings highlight glutamine-related metabolic alterations as potentially relevant to cirrhosis risk, supported by lower glutamate and glutathione levels in the preliminary LC–MS/MS analysis. Further large-scale longitudinal studies are needed to validate these findings and assess their clinical utility in diverse patient populations.

## Data Availability

The original contributions presented in the study are included in the article/Supplementary material, further inquiries can be directed to the corresponding author.
