# Supplementary material for: Exploring potential associations between blood metabolites and cirrhosis risk: a Mendelian randomization and LC–MS/MS analysis
Source: Front Med (Lausanne). 2026 Jul 2;13:1809188. doi: 10.3389/fmed.2026.1809188 (PMC13372711; doi:10.3389/fmed.2026.1809188)
Supplement: Supplementary file 1 [file Data_Sheet_1.DOCX]

***Supplementary Material***

**Supplementary Figures**

**Figure S1-S14. Sensitivity and methodological analyses for the 14 selected metabolites levels respectively.**

A leave-one-out analysis was conducted for all significant causal associations. In each panel, the black dots represent the causal association estimates between a specific exposure and the target mental disorder after sequentially discarding each SNP. The red dots represent the overall causal estimates using the random-effects inverse variance weighting method. The horizontal lines represent the 95% confidence intervals;

The scatterplot illustrates the MR analysis method employed to assess the causal effect of metabolites on cirrhosis. Each point represents an SNP, with its corresponding ratio estimate (β-IVW or β-Egger) plotted on the x-axis and the precision of the estimate (1/standard error) plotted on the y-axis. The IVW method is depicted in light blue, while the MR Egger method is shown in dark blue, with vertical lines representing their respective overall causal estimates.


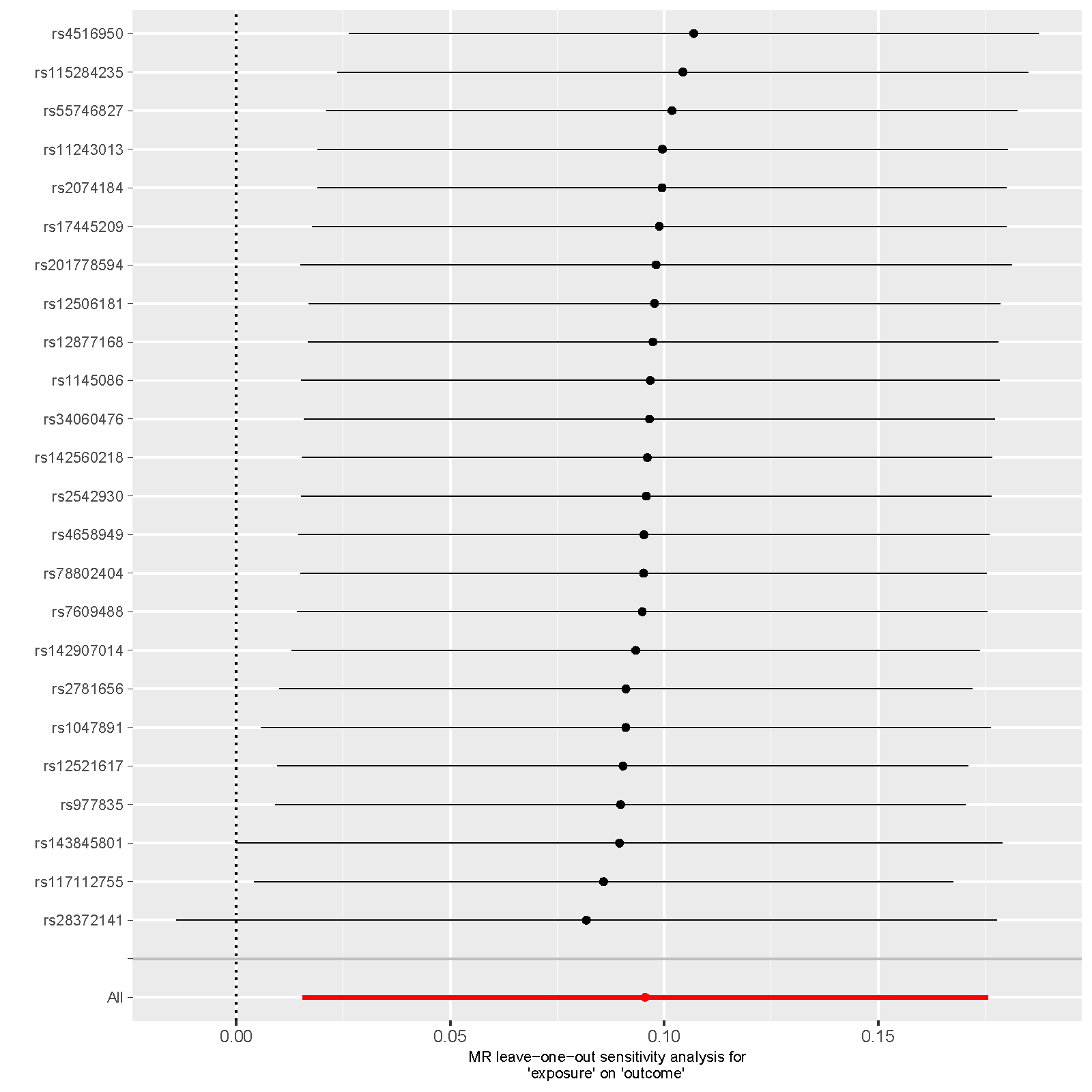

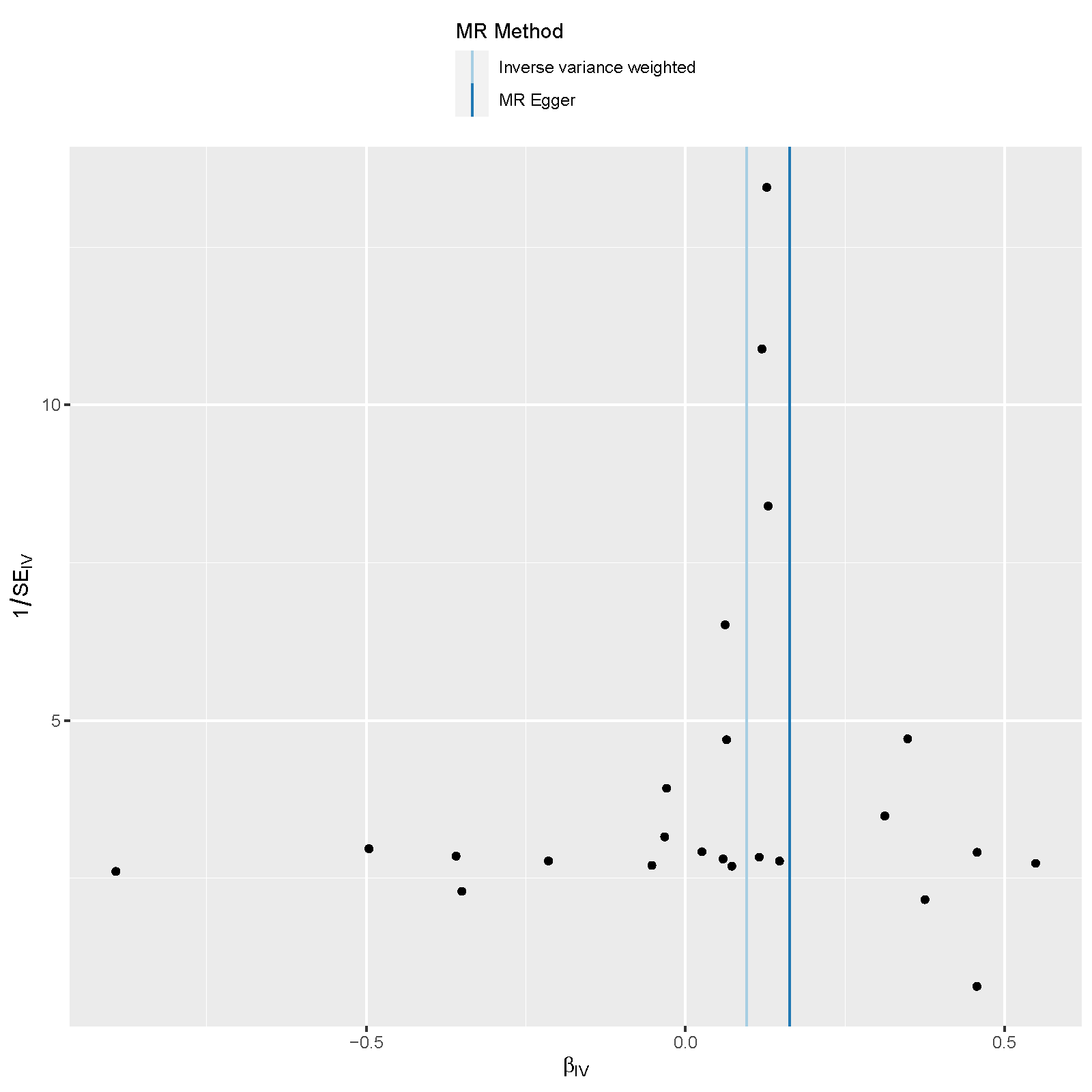


**Figure S1.** Sensitivity and methodological analyses for metabolite Creatine levels.


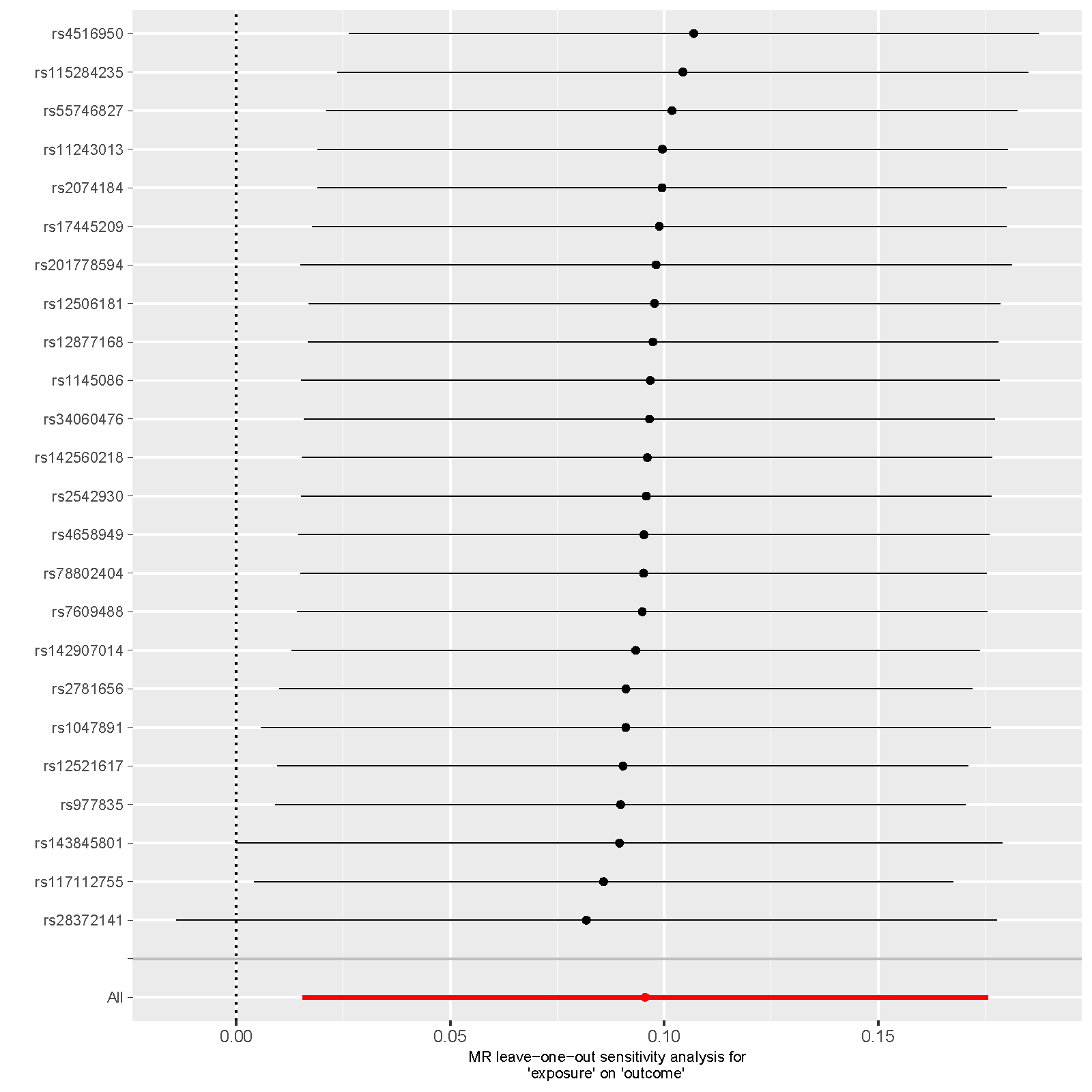

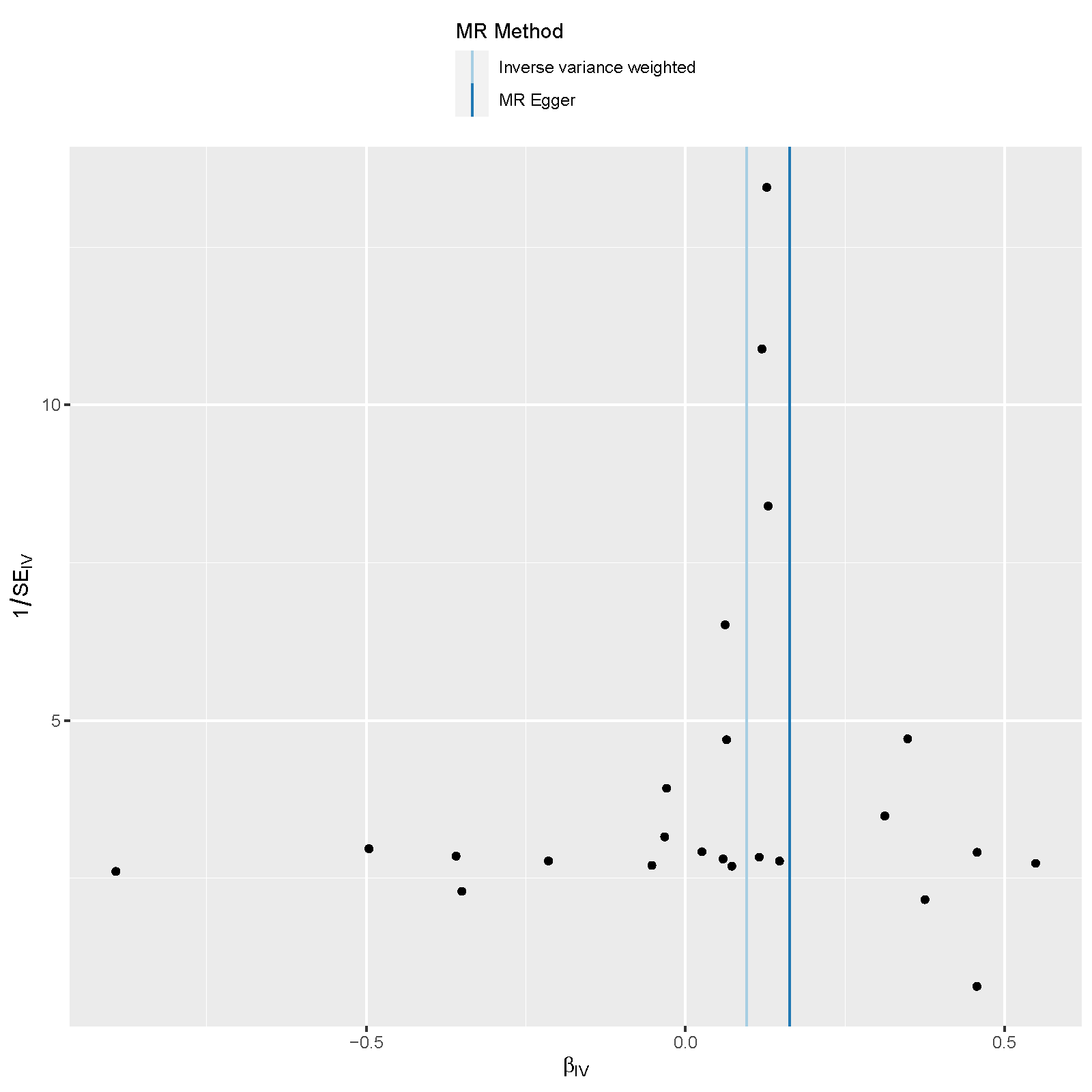


**Figure S2.** Sensitivity and methodological analyses for Propionylcarnitine (c3) levels.


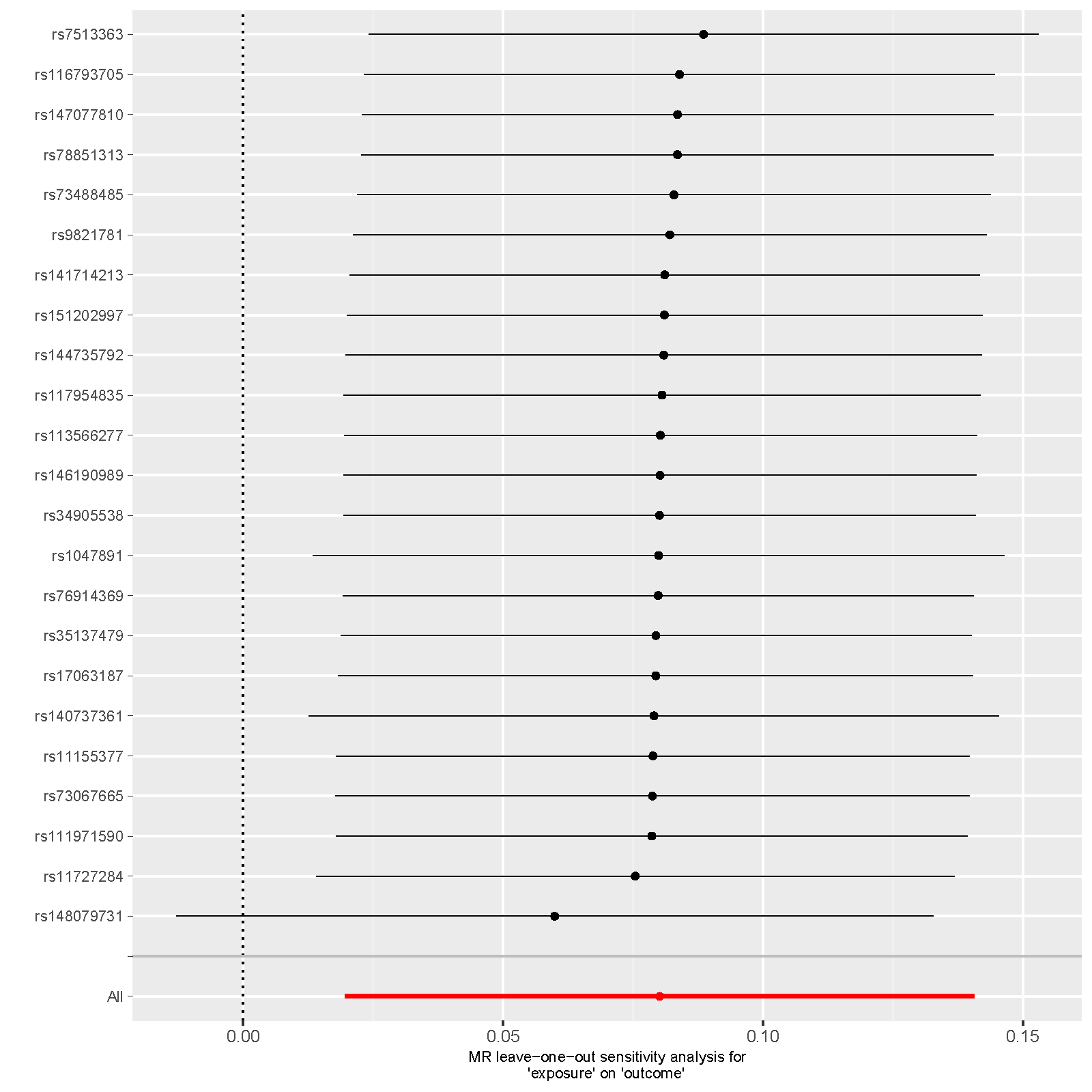

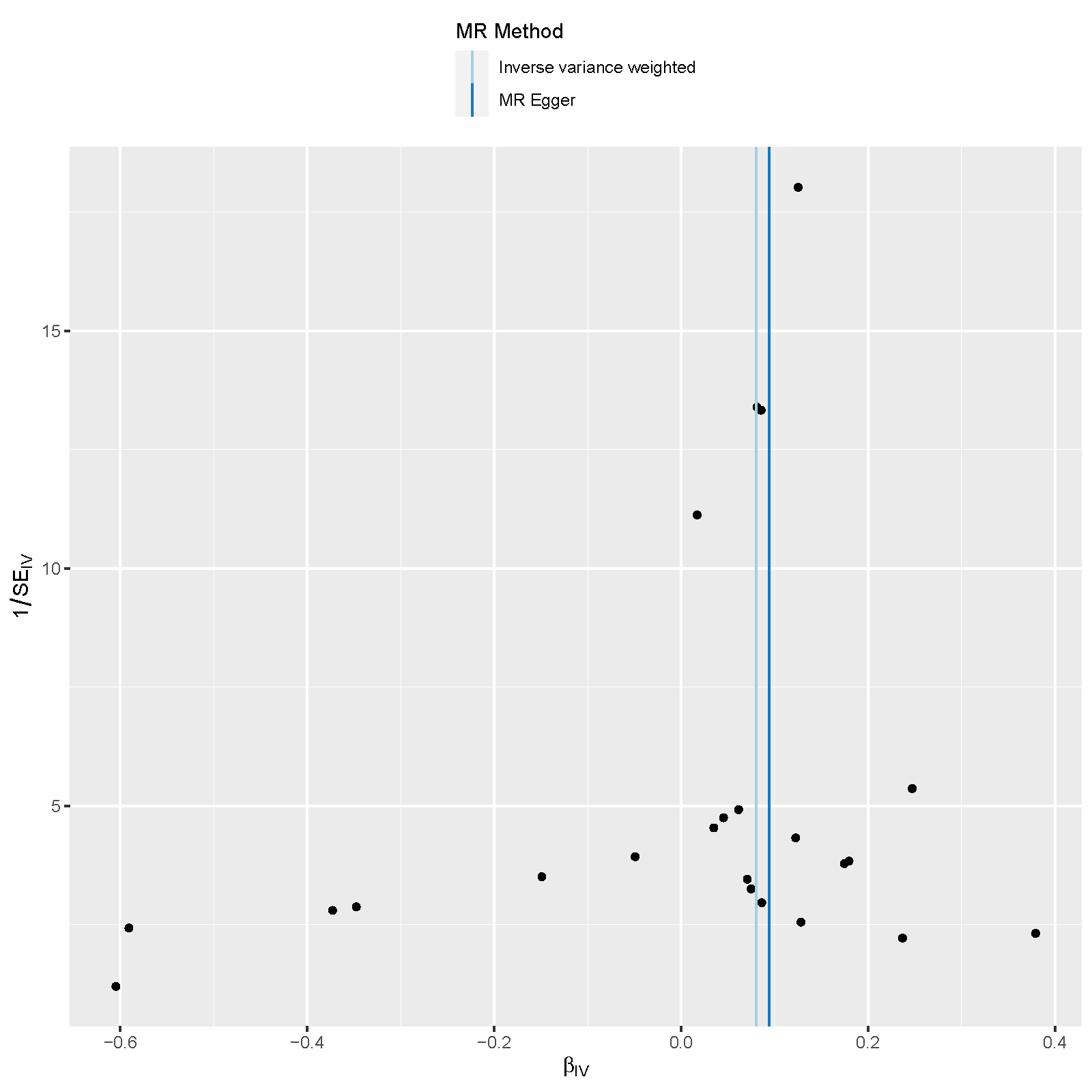


**Figure S3.** Sensitivity and methodological analyses for Hexanoylglycine levels.
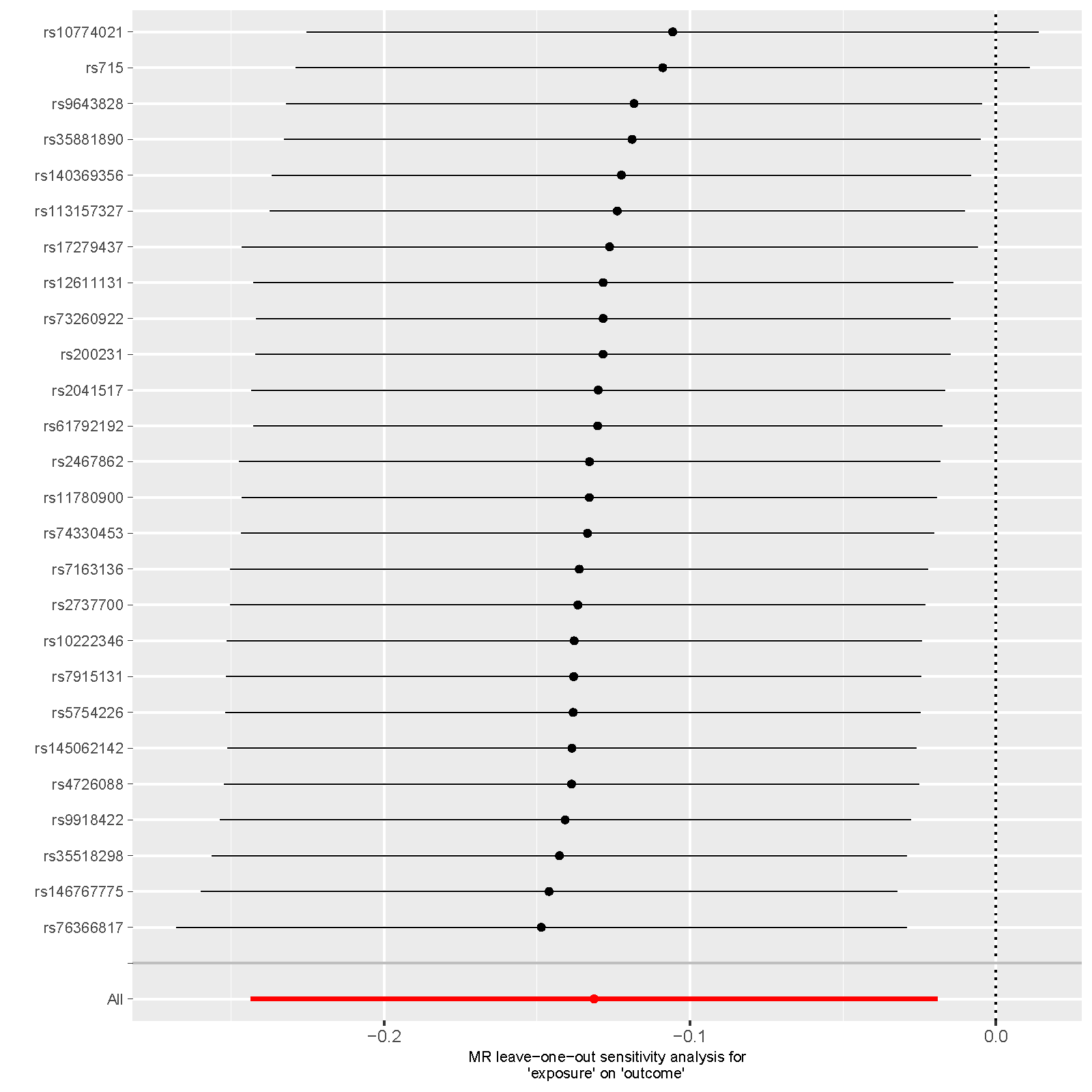

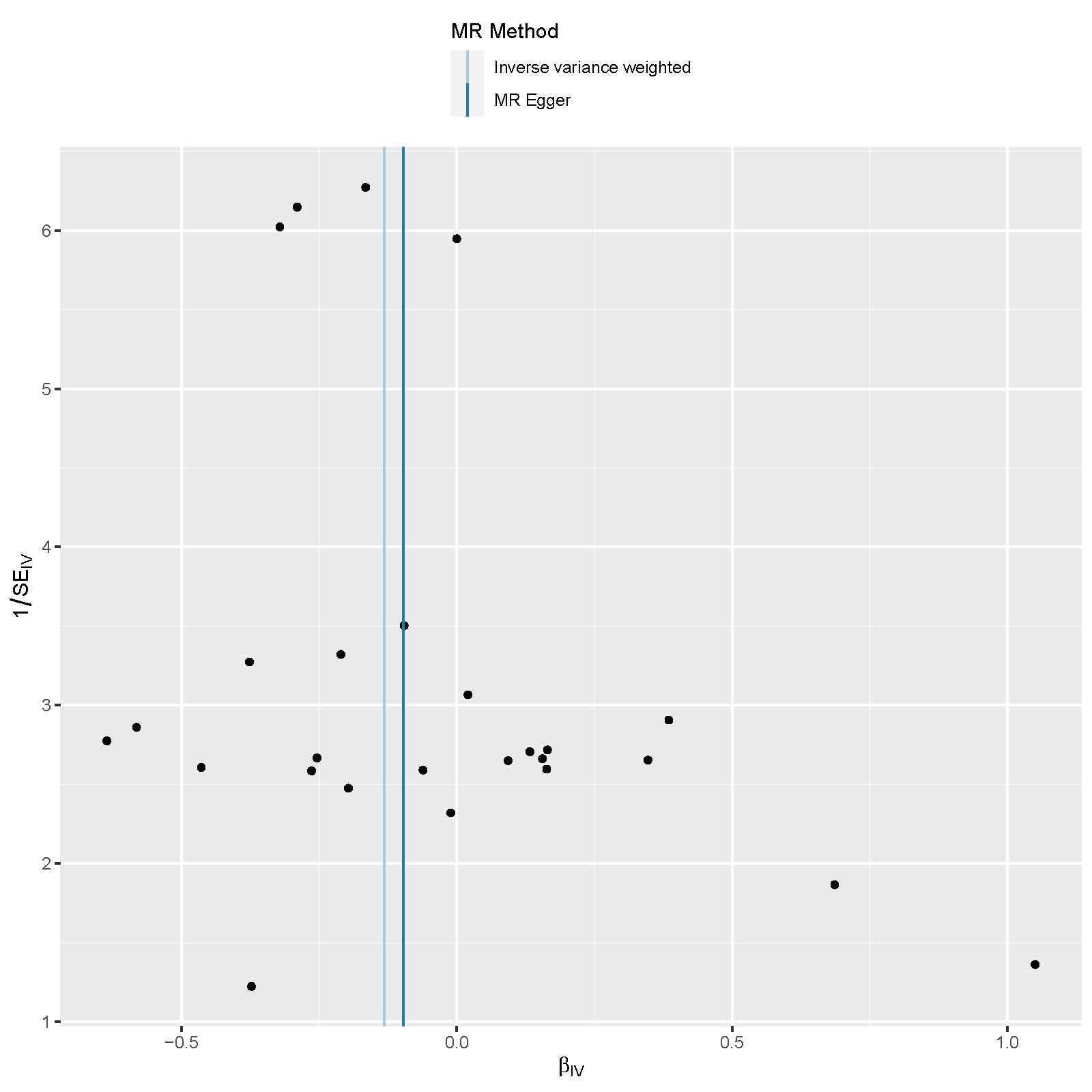


**Figure S4.** Sensitivity and methodological analyses for Glutamine degradant levels.


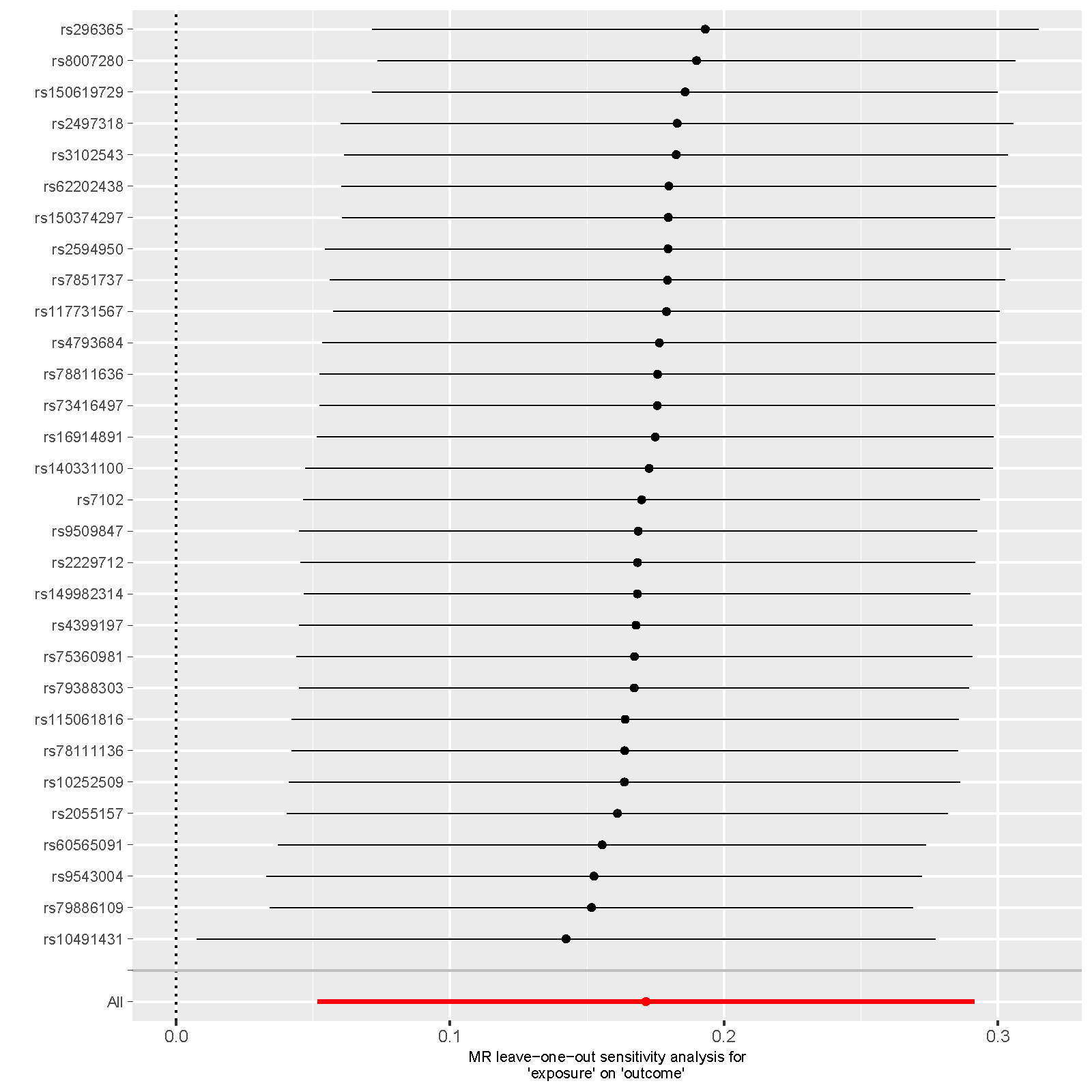

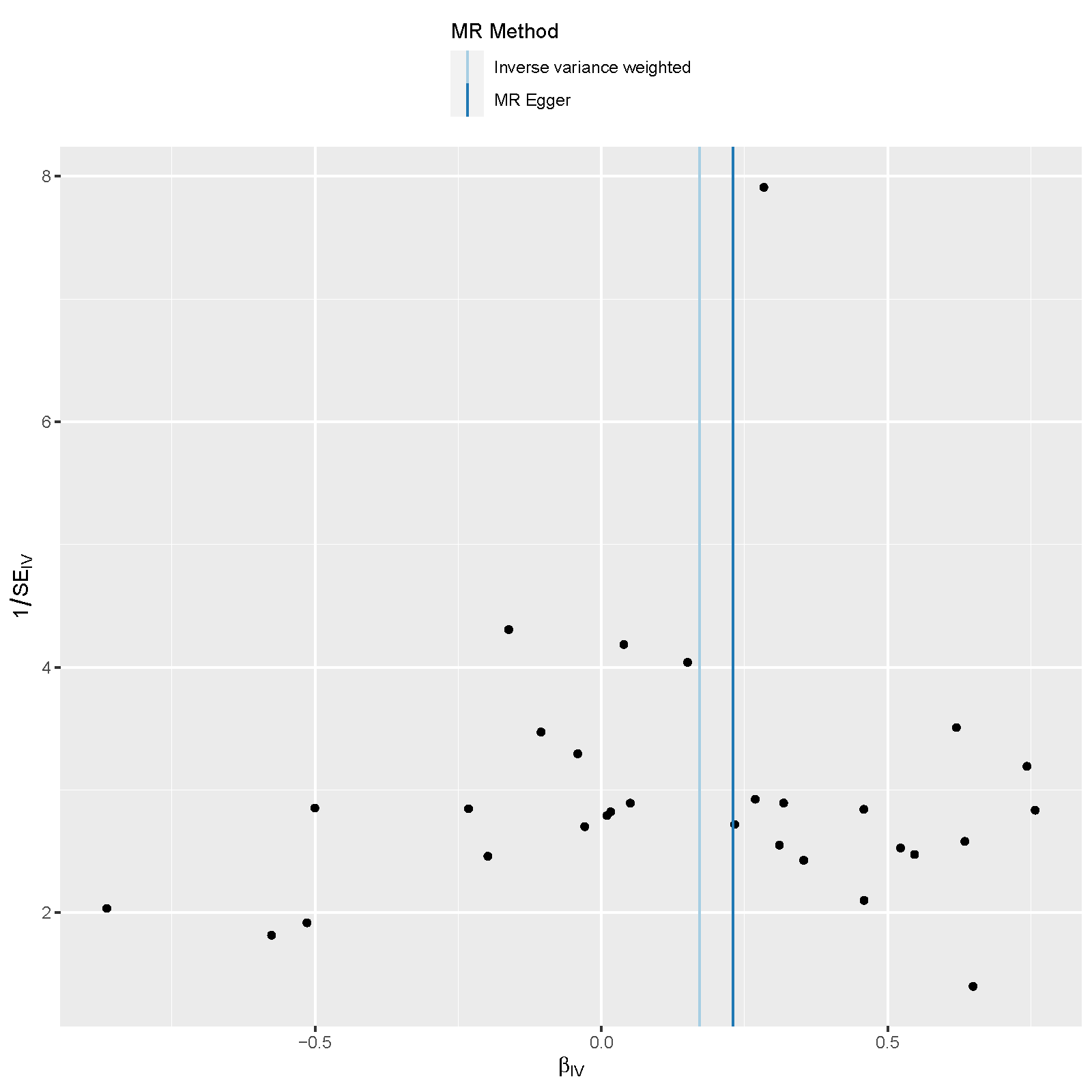


**Figure S5.** Sensitivity and methodological analyses for Pregnenediol sulfate (C21H34O5S) levels.


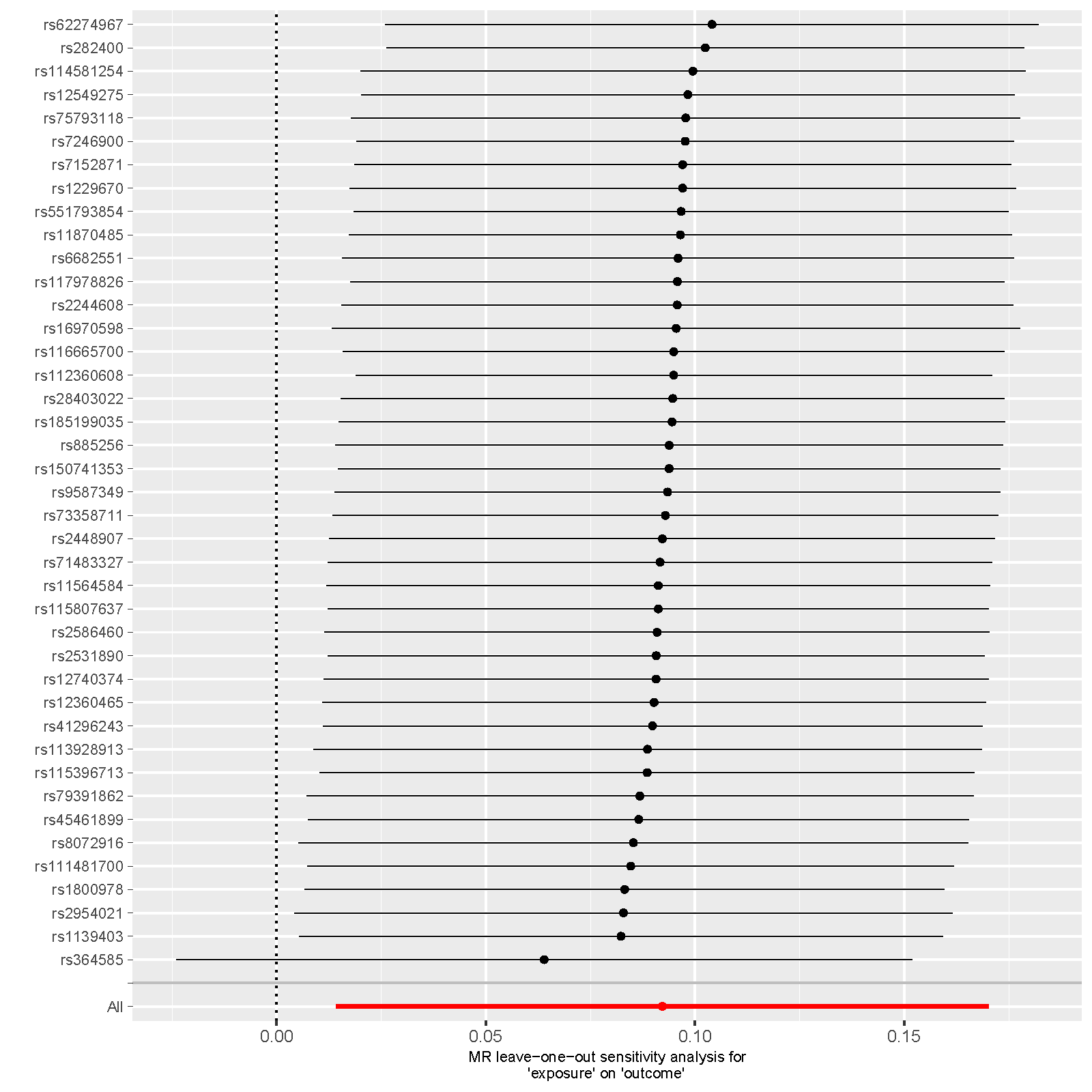

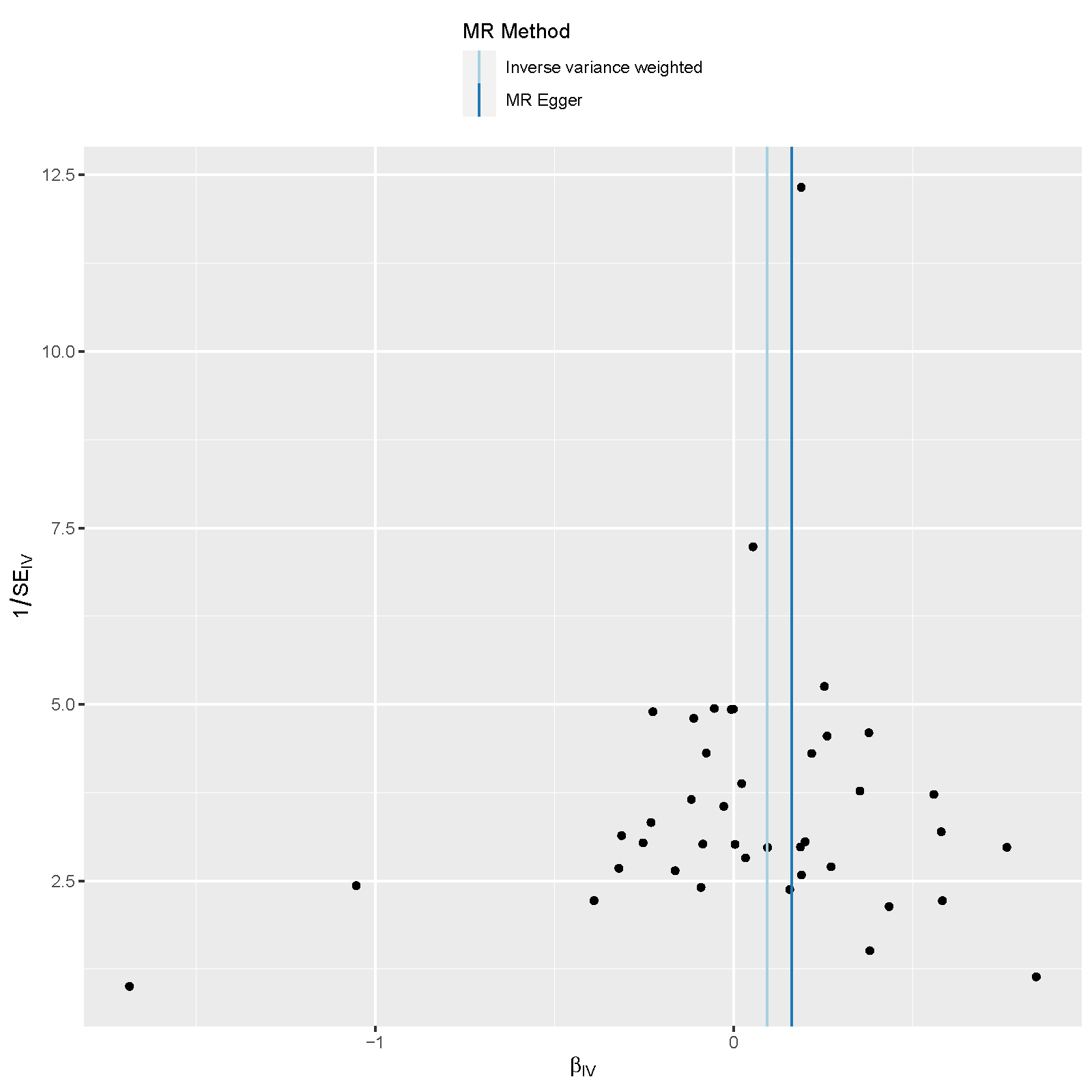


**Figure S6.** Sensitivity and methodological analyses for Sphingomyelin (d18:0/20:0, d16:0/22:0) levels.


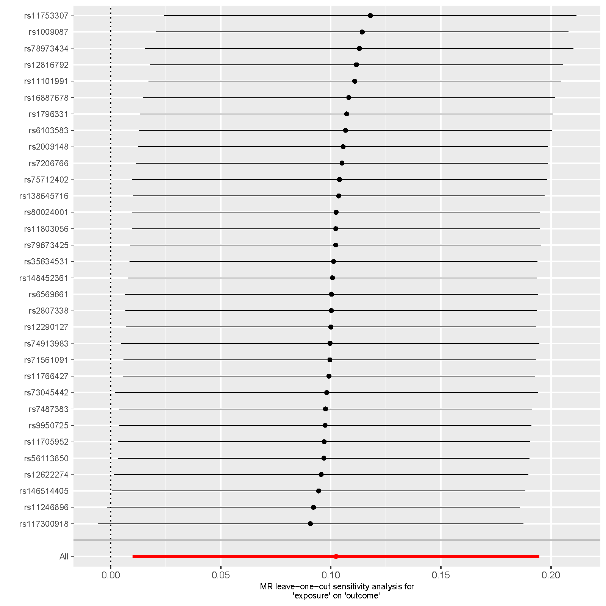

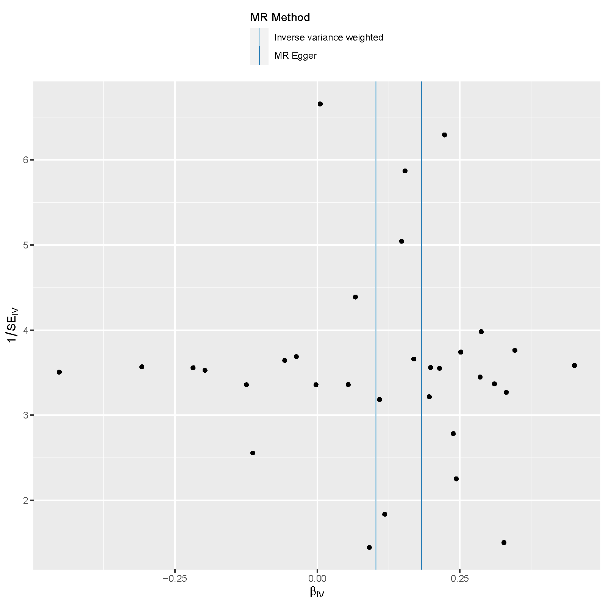


**Figure S7.** Sensitivity and methodological analyses for 3,7-dimethylurate levels.


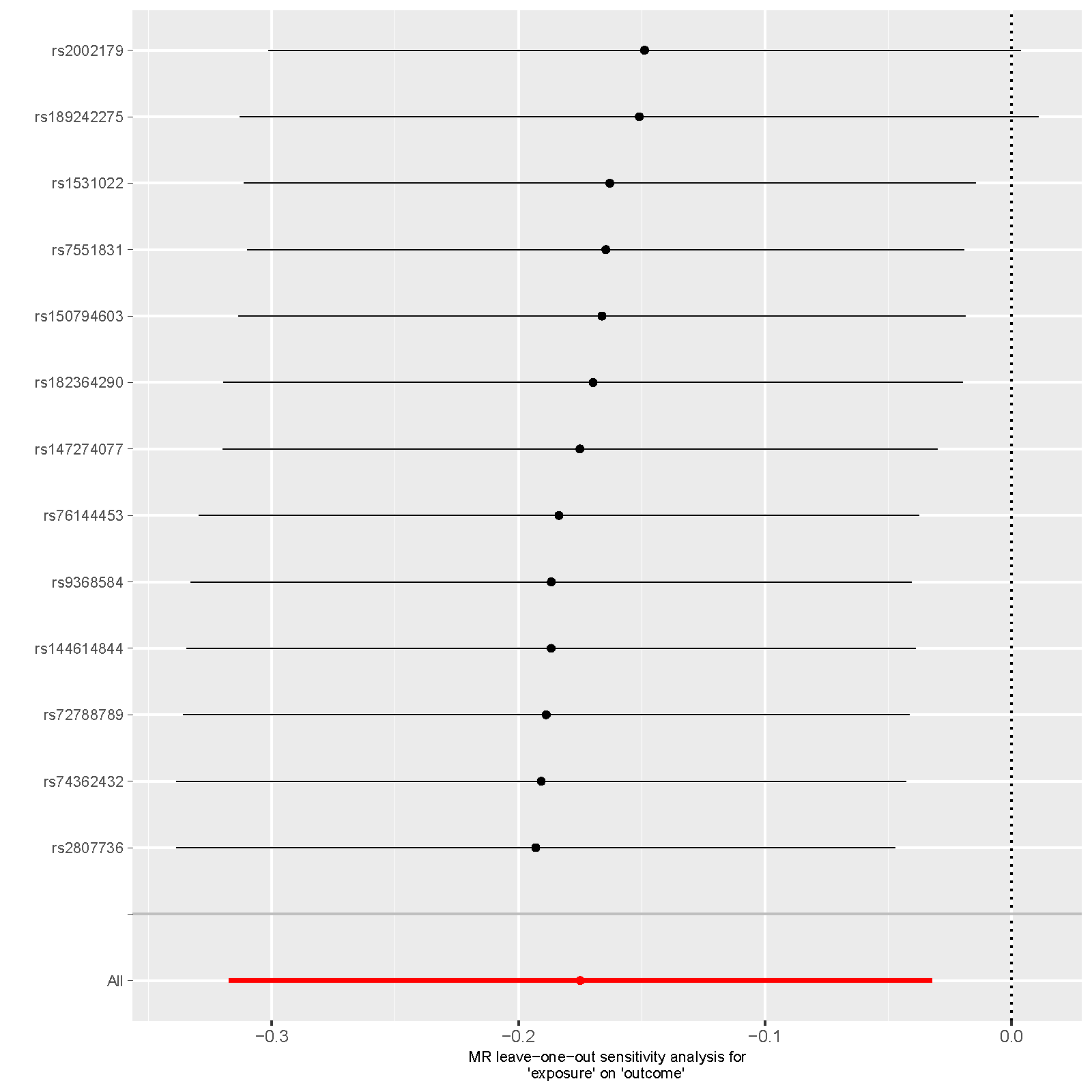

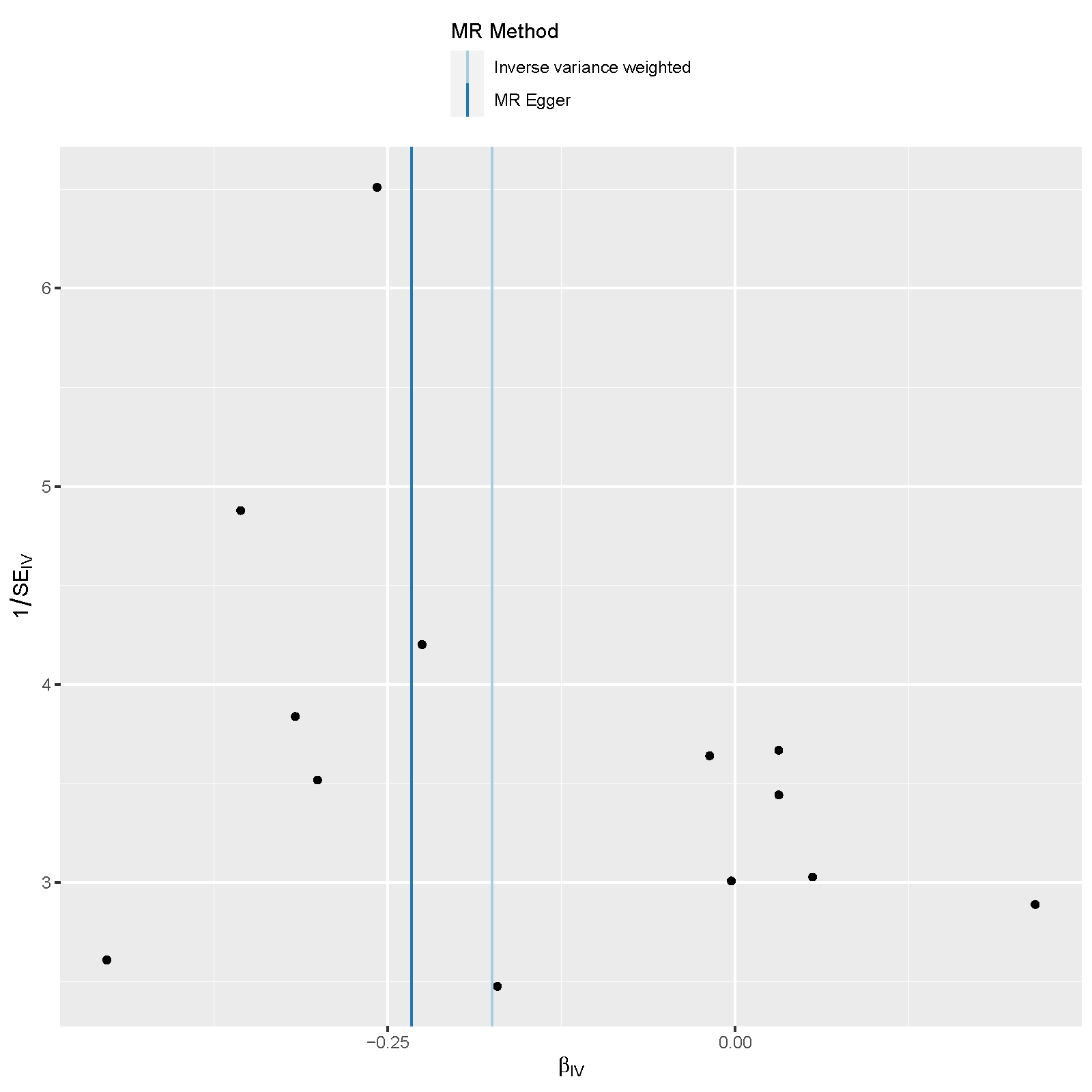


**Figure S8.** Sensitivity and methodological analyses for X-12007 levels.


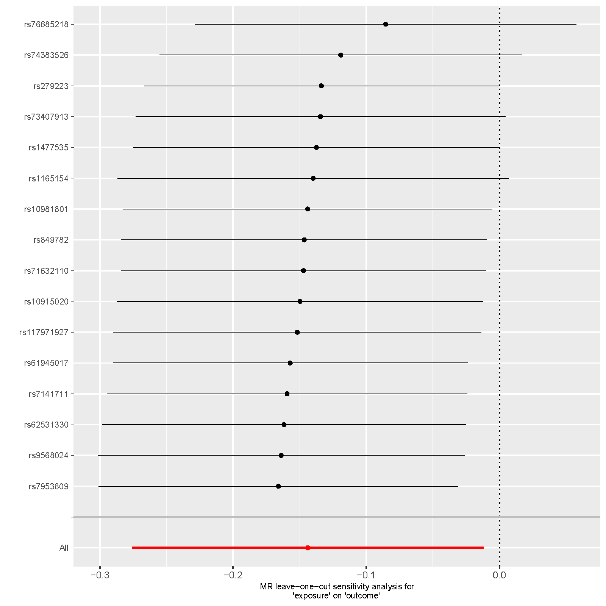

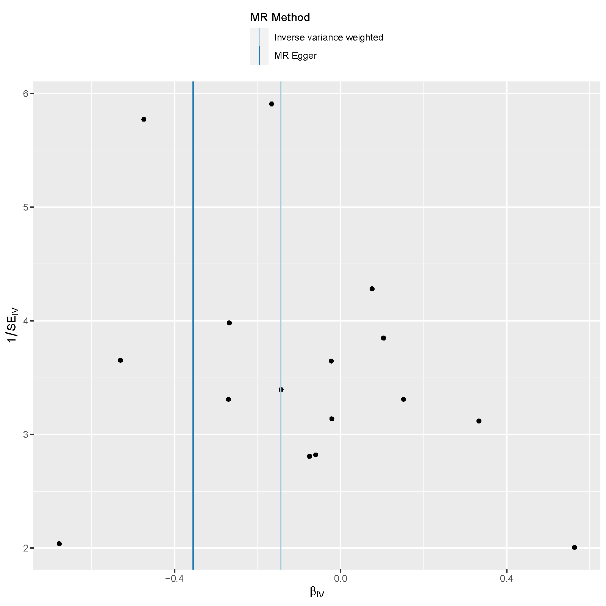


**Figure S9.** Sensitivity and methodological analyses for X-12847 levels.


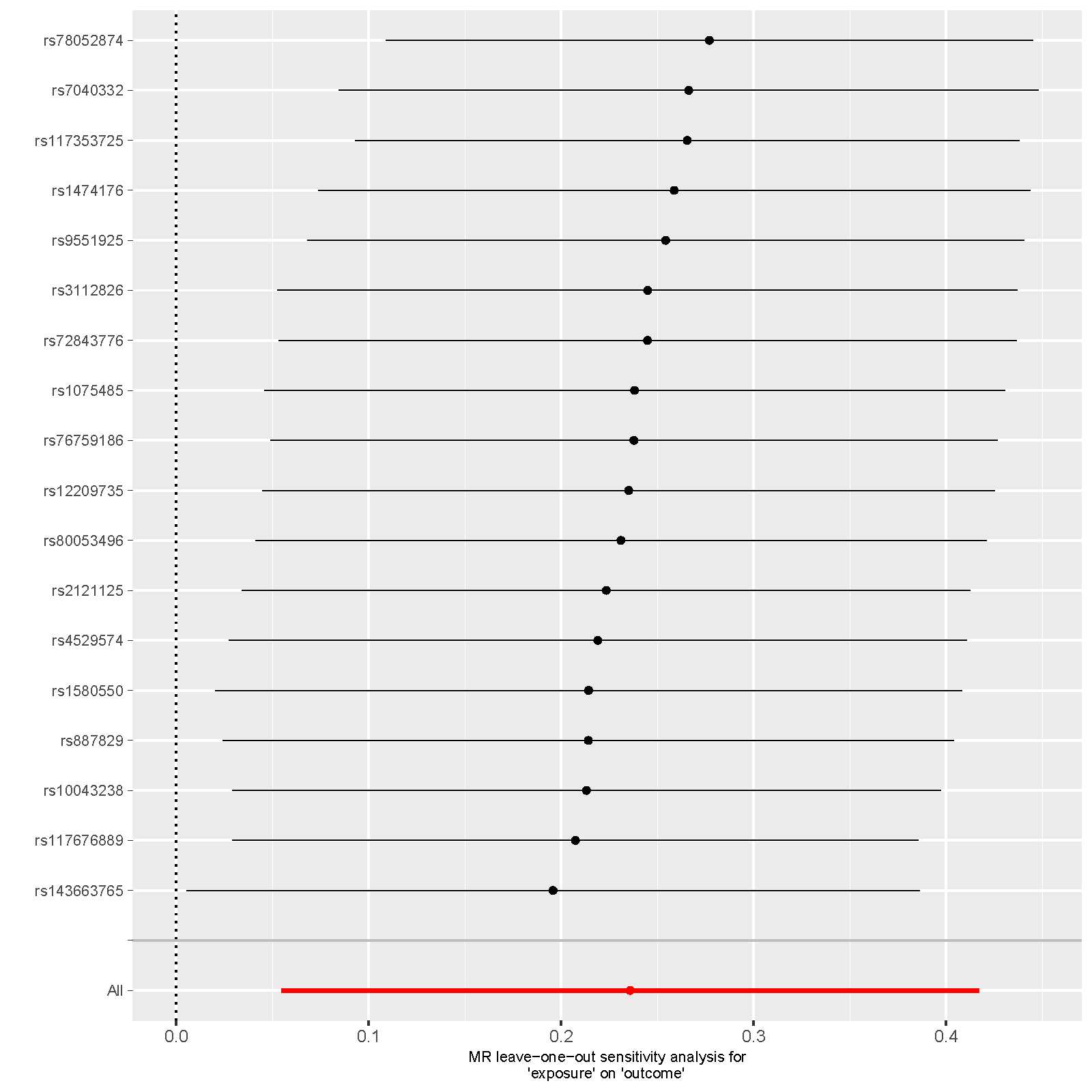

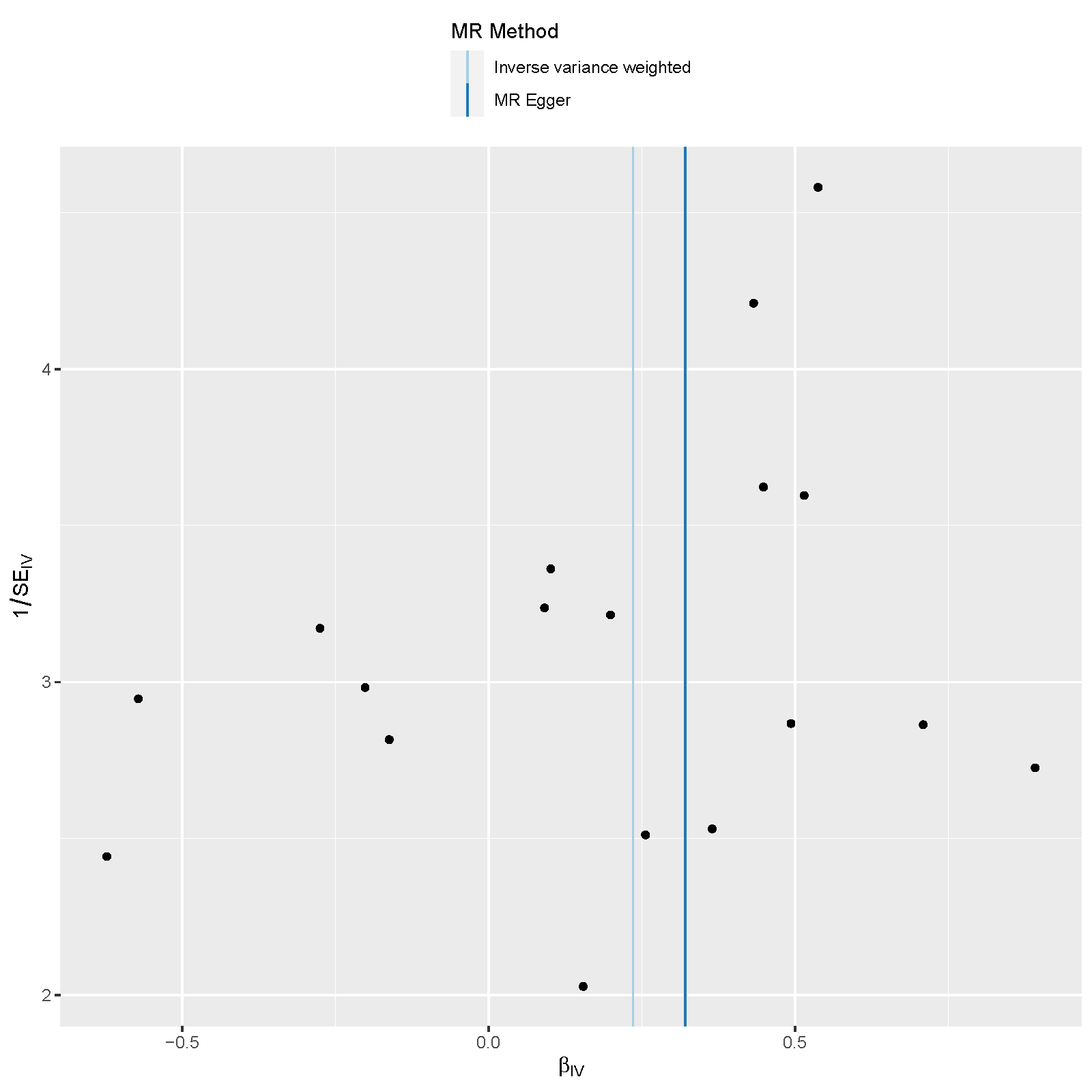


**Figure S10.** Sensitivity and methodological analyses for X-23782 levels.


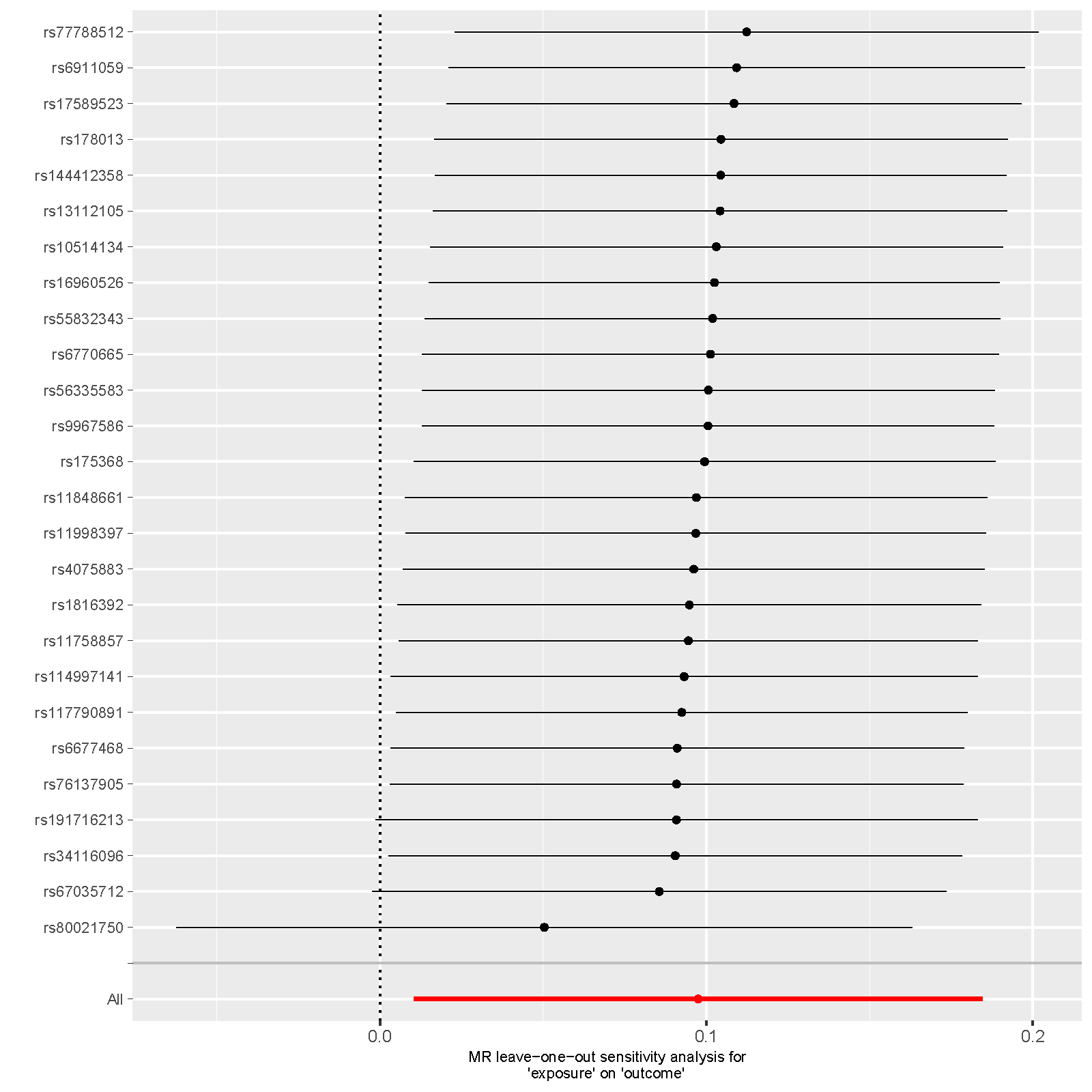

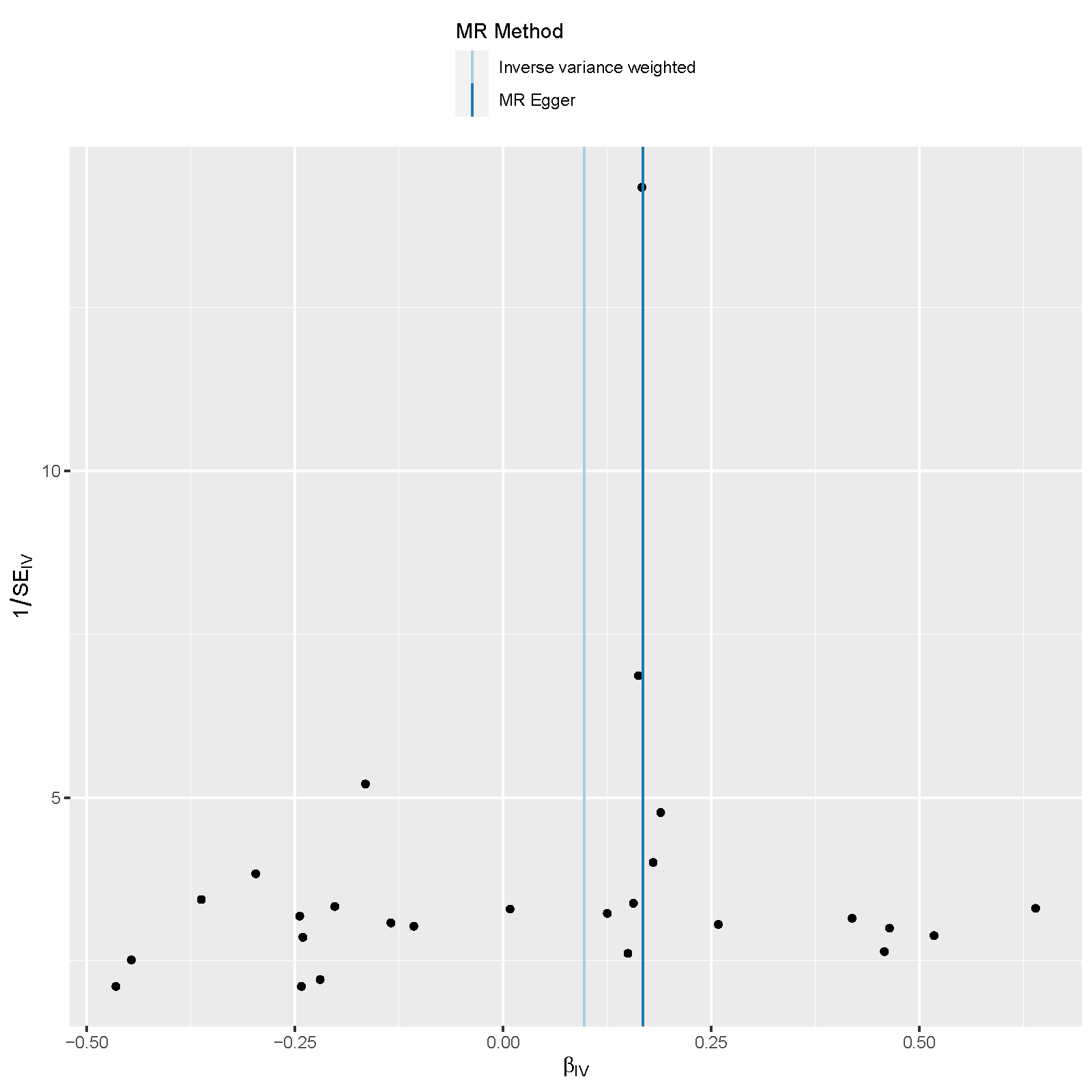


**Figure S11.** Sensitivity and methodological analyses for X-25172 levels.


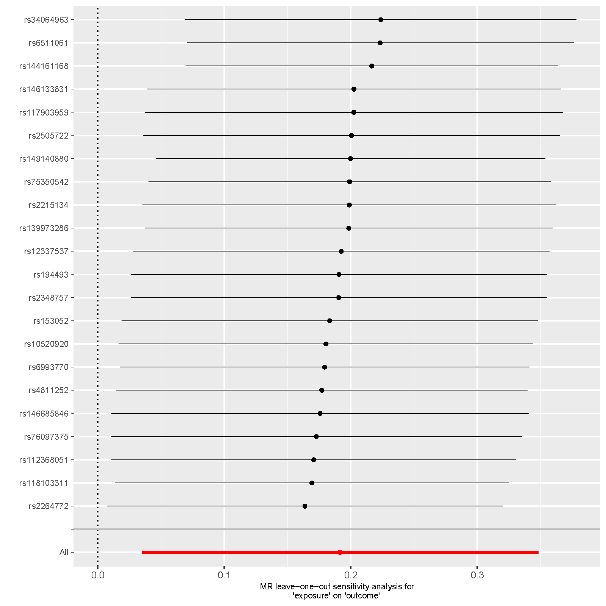

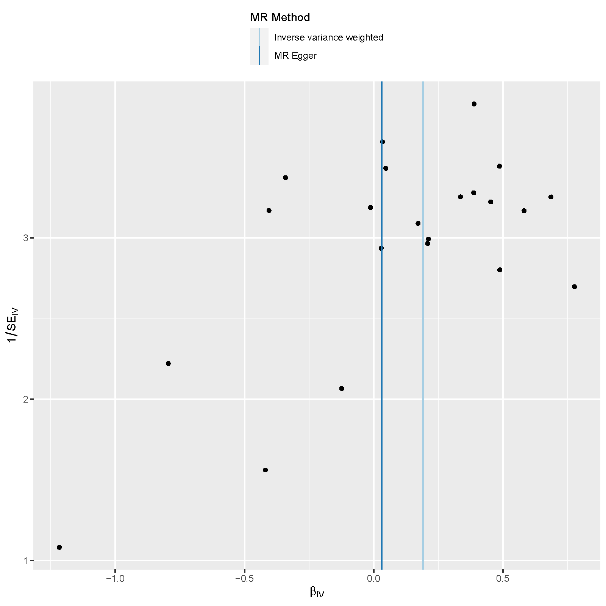


**Figure S12.** Sensitivity and methodological analyses for Adenosine 5'-monophosphate to alanine ratio.


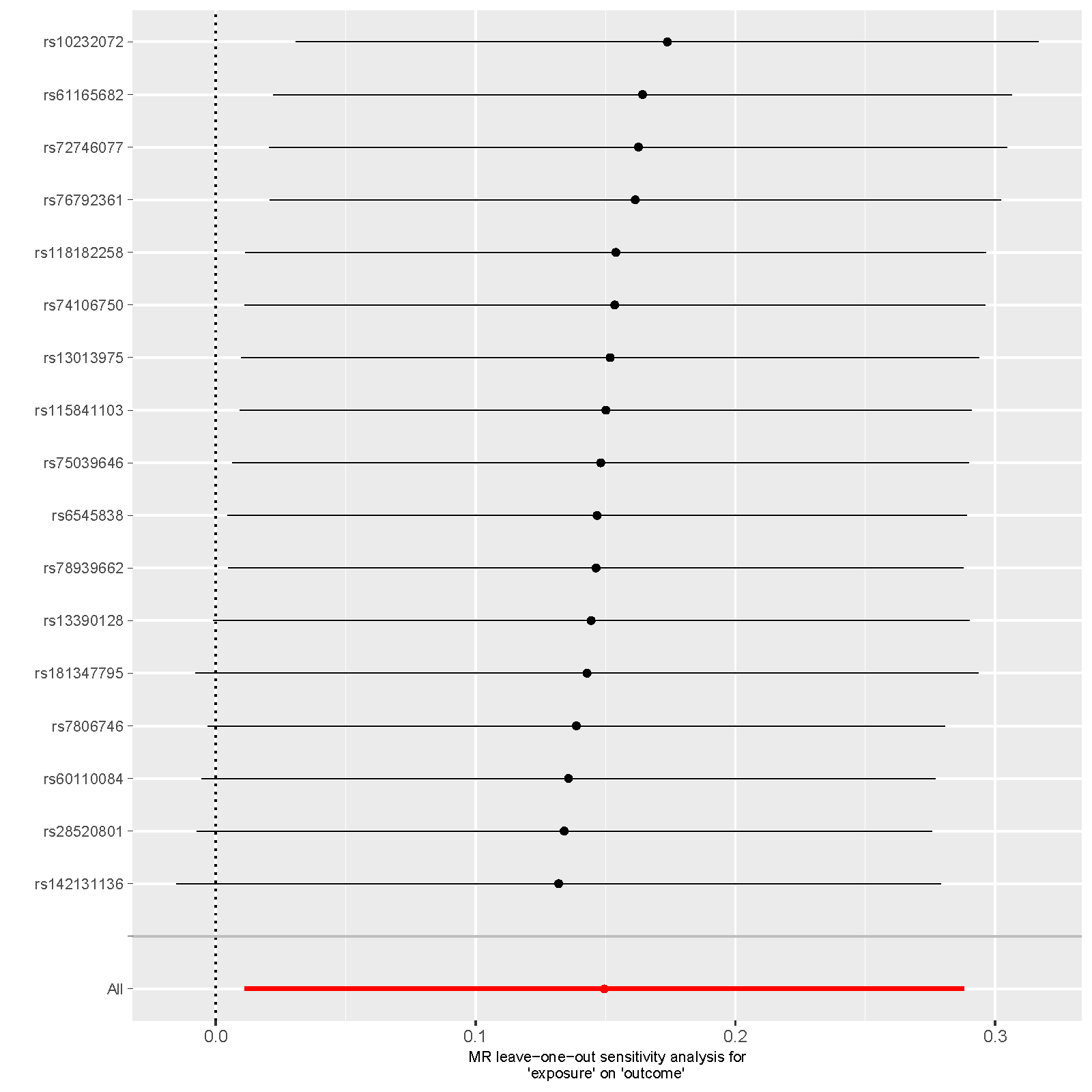

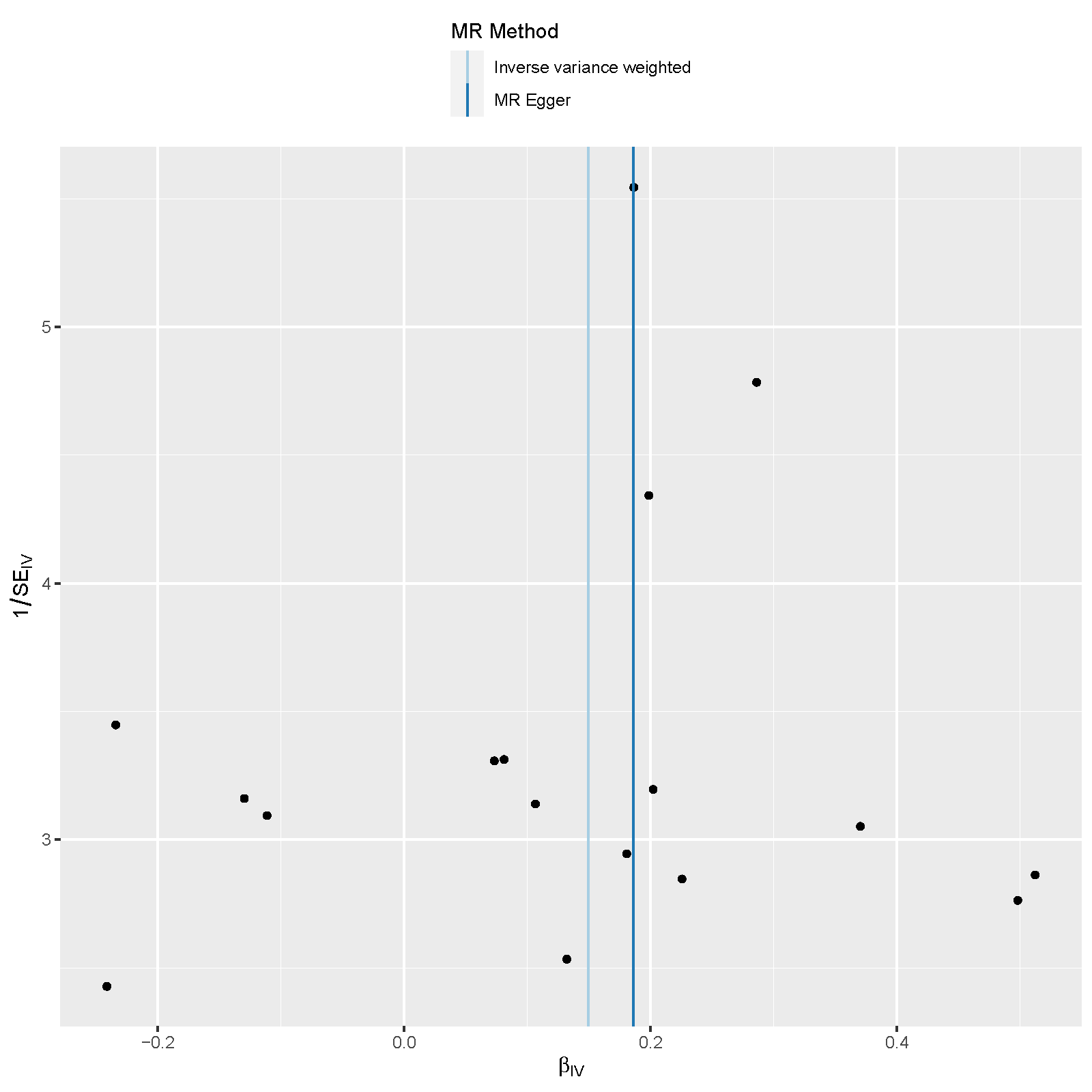


**Figure S13.** Sensitivity and methodological analyses for Adenosine 5'-monophosphate to arginine ratio.


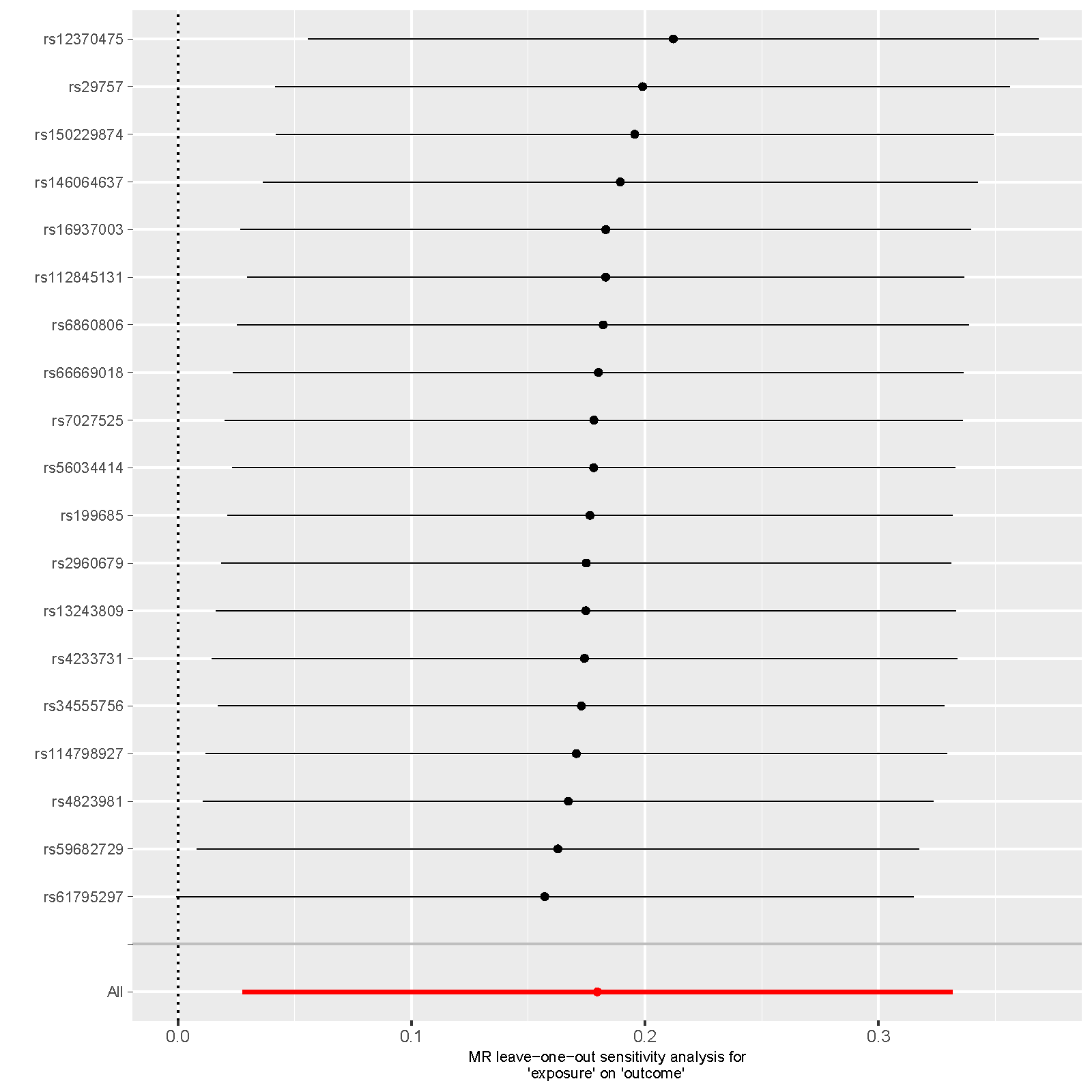

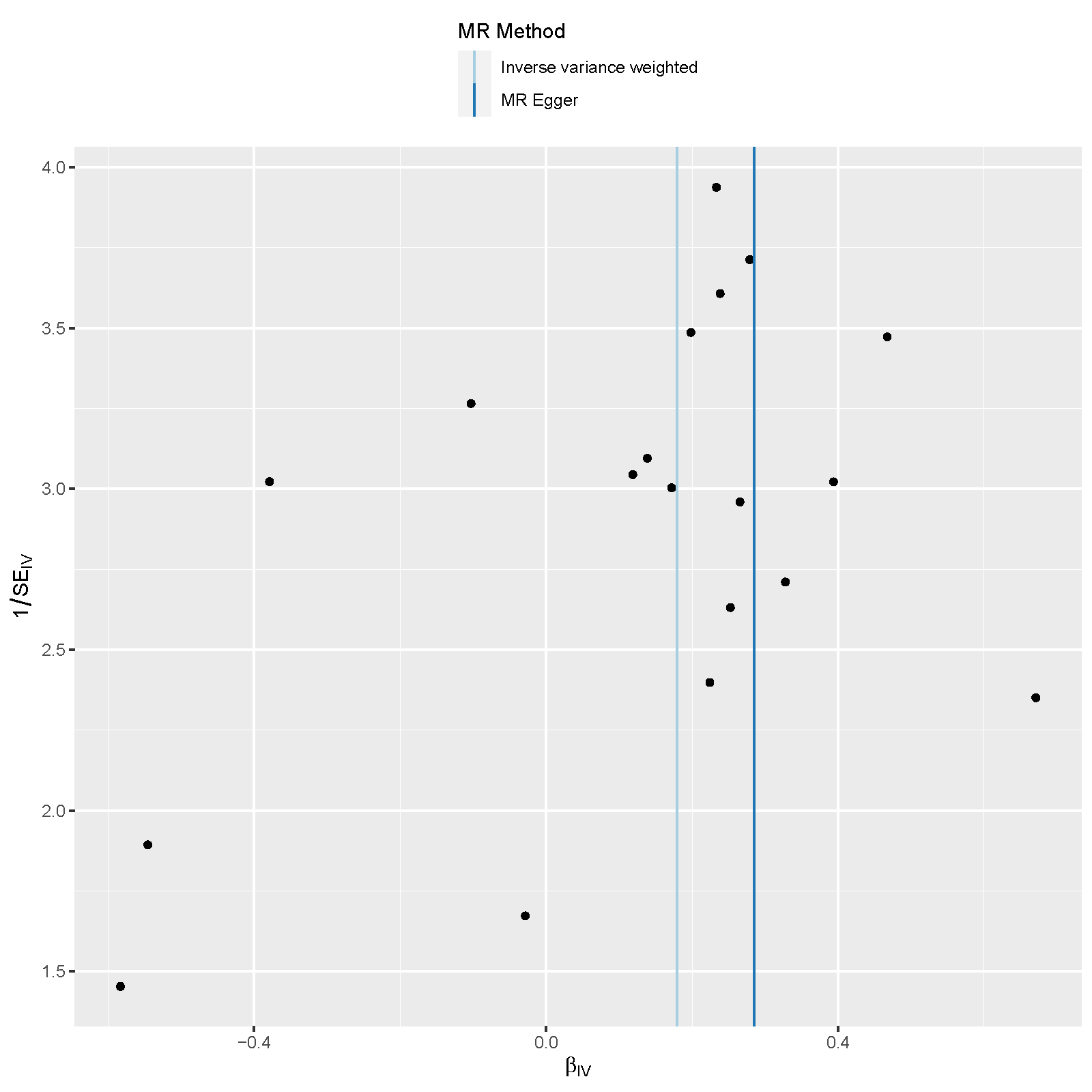


**Figure S14.** Sensitivity and methodological analyses for Carnitine to acetylcarnitine (C2) ratio.
