# Supplementary material for: Exploring potential associations between blood metabolites and cirrhosis risk: a Mendelian randomization and LC–MS/MS analysis
Source: Front Med (Lausanne). 2026 Jul 2;13:1809188. doi: 10.3389/fmed.2026.1809188 (PMC13372711; doi:10.3389/fmed.2026.1809188)
Supplement: Supplementary file 2 [file Data_Sheet_2.DOCX]

**
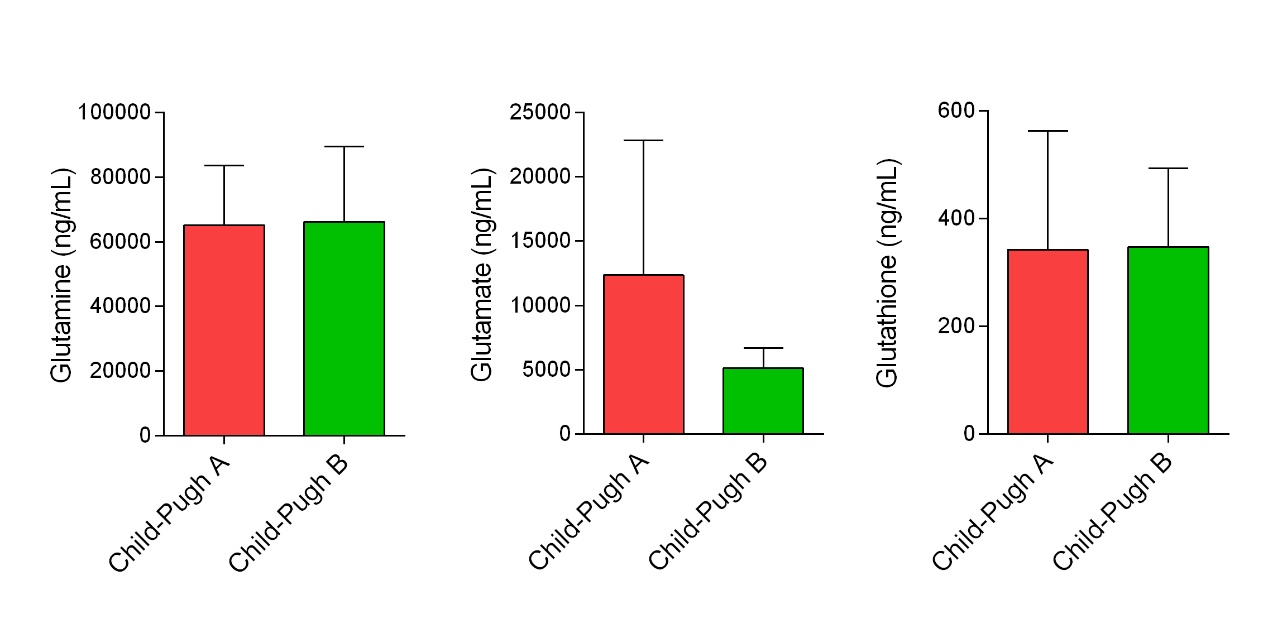
**

**Figure S15.** Descriptive analysis of glutamine and its metabolite concentration levels in plasma between patients with Child-Pugh and B. Glutamine, glutamate, and glutathione levels were compared descriptively between patients with Child-Pugh A (n = 7) and B (n = 3). No formal statistical testing was performed because of the limited sample size.
