# Supplementary material for: Exploring potential associations between blood metabolites and cirrhosis risk: a Mendelian randomization and LC–MS/MS analysis
Source: Front Med (Lausanne). 2026 Jul 2;13:1809188. doi: 10.3389/fmed.2026.1809188 (PMC13372711; doi:10.3389/fmed.2026.1809188)
Supplement: Supplementary file 3 [file Table_1.DOCX]

***Supplementary Material***

**Supplementary Table**

**Table S1. Baseline characteristics of control and cirrhosis patients.**

|  | **Control (n=10)** | **Cirrhosis (n=10)** | **P value** |
| --- | --- | --- | --- |
| Age, years | 34(31-43) | 47(44-52) | < 0.001 |
| Male patients, n (%) | 6(60) | 7(70) | 0.639 |
| TB, μmol/L | 10.8(10-28) | 27.6(23.7-53.8) | < 0.001 |
| DB, μmol/L | 3.90(3.6-7.2) | 14.0(7.8-24.8) | < 0.001 |
| ALT, U/L | 24.5(21-32) | 27.5(25-61) | 0.105 |
| AST, U/L | 24.5(22-29) | 33(29-44) | 0.005 |
| ALB, g/L | 46.5(42.6-47.1) | 41.0(38.3-44.4) | 0.089 |
| GGT, U/L | 22.5(17-32) | 68(39-117) | 0.006 |
| ALP, U/L | 66(53-72) | 99(65-165) | 0.04 |
| Platelet×10^9/L | 161(123-210) | 85(62-99) | < 0.001 |
| PT, s | 9.5(7.3-10.3) | 11(9.6-14.2) | 0.019 |
| LSM, kPa | 6.2(4.9-6.6) | 17(14.8-19.9) | < 0.001 |
| Portal hypertension | 0 | 3 | 0.010 |
| Child-Pugh classification |  |  | < 0.001 |
| A | 0 | 7 |  |
| B | 0 | 3 |  |
| C | 0 | 0 |  |

ALT, Alanine aminotransferase; AST, Aspartate aminotransferase; TB, total bilirubin; DB, direct bilirubin; TG, triacylglycerol; GGT, gamma glutamyltransferase; ALP, alkaline phosphatase; LSM, liver stiffness measurement.
